# Traceable and Scalable Food Balance Sheets from Agricultural Commodity Supply and Utilization Accounts (2010–2022)

**DOI:** 10.1038/s41597-025-05137-y

**Published:** 2025-05-21

**Authors:** Xin Zhao, Maksym Chepeliev, Neus Escobar, Matthew T. Binsted, Pralit Patel, Page Kyle, Marshall A. Wise

**Affiliations:** 1 Joint Global Change Research Institute, Pacific Northwest National Laboratory, 5825 University Research Ct, College Park, MD 20740 USA; 2 Center for Global Sustainability, School of Public Policy, Thurgood Marshall Hall, University of Maryland, College Park, MD 20740 USA; 3 Center for Global Trade Analysis, Department of Agricultural Economics, Purdue University, 403 Mitch Daniels Blvd, West Lafayette, IN 47906 USA; 4 Basque Centre for Climate Change (BC3), Scientific Campus of the University of the Basque Country, 48940 Leioa, Spain; 5 Biodiversity and Natural Resources (BNR) Program, International Institute for Applied Systems Analysis (IIASA), Schlossplatz 1, 2361 Laxenburg, Austria

**Keywords:** Environmental social sciences, Agriculture, Interdisciplinary studies

## Abstract

The Food Balance Sheets (FBS), compiled by the Food and Agriculture Organization (FAO), serve as a cornerstone dataset for studies on agricultural development, food security, and dietary health, providing a broad overview of global and regional food systems. However, its limited transparency and scalability hinder its application in empirical analysis and multisector dynamic modeling. Here, we present a traceable Food Balance Sheets (T-FBS) dataset, developed from detailed Supply Utilization Accounts (SUA) using a novel Primary Commodity equivalent (PCe) aggregation approach. This framework enables the aggregation of commodity flows along supply chains while ensuring consistency and balance across multiple dimensions. The T-FBS dataset includes 57 PCe commodities across 195 regions for the period 2010–2022, consolidated from over 500 SUA products. While T-FBS closely aligns with FAO-FBS at aggregate levels for dietary energy and macronutrients, it identifies key uncertainties in other elements (e.g., feed, trade, stocks). By enhancing methodological transparency, traceability, and scalability, T-FBS strengthens the robustness of food system studies and fosters future research and collaboration within the open-source community.

## Background & Summary

The Food Balance Sheets (FBS), compiled by the Food and Agriculture Organization (FAO) of the United Nations, serve as a foundational dataset for thousands of studies on agricultural development, global value chains, food security, dietary health, and the Sustainable Development Goals^1–8^. It provides a detailed representation of a country’s production, international trade (imports and exports), utilization (e.g., food, feed, tourist consumption, processing, seed, losses, other non-food uses), and stock variation, as well as dietary energy (calories) and macronutrient (e.g., fats and proteins) availability for about 100 aggregated commodity groups, encompassing the diversity of food products^9,10^. The FBS offers a comprehensive overview of global and regional food systems^11^. While the FBS dataset is openly accessible^11^, the methodology and assumptions underlying its compilation are not fully transparent, limiting its traceability and reproducibility.

The FBS is compiled and prepared by FAO’s Statistics Division (FAOSTAT; https://www.fao.org/faostat) based on a more detailed dataset, the Supply Utilization Accounts (SUA)^12^. Historically, FAOSTAT released an older version of the FBS covering the period 1961–2013, after which updates ceased. More recently, FAOSTAT developed an updated methodology^10^ to compile a new version of the FBS (released around 2019), providing data from 2010 onwards (now extending to 2022)^13^. Recent studies have highlighted key differences both between the two versions of the dataset and between FBS and other sources (e.g., household budget surveys), emphasizing the need for greater transparency in their compilation and application^14–16^.

The release of the new FBS was accompanied by the inclusion of the SUA, covering the same period (i.e., SUA prior to 2010 remains unavailable). Approximately 500 agri-food commodities in SUA are aggregated into 85 crop and livestock products in the FBS (hereinafter referred to as FAO-FBS), along with a dozen fisheries and aquaculture products (compiled separately). Notably, the aggregation of SUA into larger groups in the FAO-FBS goes beyond the simple summation of commodities mapped^17^.

The compilation of FBS involves the conversion of various food products into their Primary Commodity equivalents (PCe)^9^, using additional data such as extraction rates and transformation coefficients, which are not publicly available. Additional adjustments or assumptions are needed when aggregating supply chains across major commodities to avoid double counting, particularly when multiple co-products are derived from the same input or when multiple inputs are processed into a single product. Further procedures are implemented to ensure the balance between imports and exports, opening and closing stocks, and total supply and demand across years and regions. However, the FAO has not yet provided a complete and traceable methodology that would enable the replication of the FBS from the underlying SUA.

The PCe approach aims to convert quantities of all processed products into their primary crop equivalents, considering the specific supply chains involved and underlying conversion efficiencies. For example, a crop equivalent is defined as the amount of primary crops embedded in the flows of derivatives that are ultimately generated at the end of each supply chain^18^. Calculating this for all commodities in the SUA is considerably more complex than other equivalence conversion methods, such as carbon dioxide equivalent (CO_2_e), gasoline gallon equivalent (GGE), or full-time equivalent (FTE), which function as simple unit conversions using scaling factors.

Unlike financial balance sheets, which balance assets against liabilities and equity, compiling the FBS using the PCe approach involves unique challenges. Specifically, it requires maintaining consistency across multiple dimensions characterizing product flows: (1) the country of origin and destination, (2) the supply chain configuration (i.e., linkages between inputs and outputs), (3) the point of processing (i.e., the country in which inputs are transformed into derivatives), and (4) the time of processing (i.e., processing may occur in one year with products carried forward to the next year in stocks). Additional considerations may be needed to preserve the relationship between primary production and inputs (e.g., land), ensuring consistent activity and productivity indicators, as well as the linkages between food availability and dietary energy or macronutrient content.

**Fig. 1:**
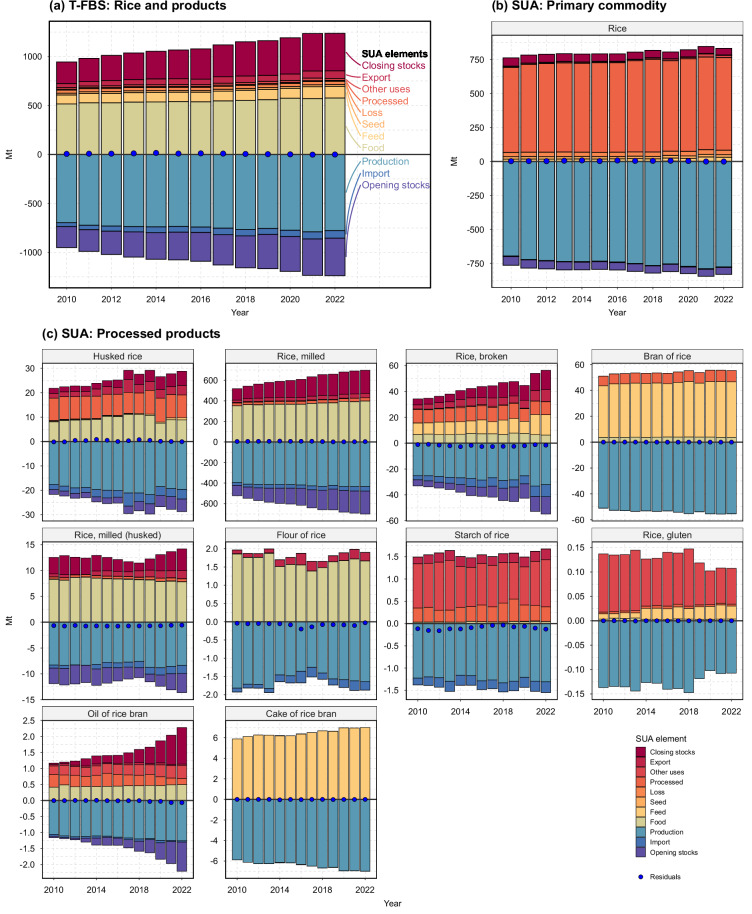
Global supply utilization balances for rice and products in Primary Commodity equivalents (PCe) from the traceable FBS (T-FBS) and for corresponding primary and processed products from the SUA. Panel (**a**) illustrates the supply utilization balances in PCe from the T-FBS, while Panels (**b,c**) show the balances for the corresponding primary product (rice, paddy) and processed products, respectively. Regional supply (negative values, shown as filled color bars) and regional demand (positive values, shown as filled color bars) are displayed for all commodities from 2010 to 2022 at the world level (covering 195 regions). Gross trade values are included, with gross exports equaling gross imports annually at the world level. Residuals, representing the difference between regional supply and demand, are indicated by points.

This study aims to introduce a transparent, reproducible, and consistent PCe approach for aggregating commodity flows along the supply chain. The approach is also scalable, accommodating varying supply chain lengths, and applicable to any commodity group. Using this approach, we develop a new traceable FBS dataset (hereafter referred to as T-FBS) by aggregating SUA using an established R package framework (*gcamfaostat v1.1*)^19^ (Methods). As an illustration, Fig. 1a presents global supply utilization balances for “rice and products” in PCe from the T-FBS, compiled by aggregating the corresponding primary (Fig. 1b) and processed products (Fig. 1c) from the SUA using the PCe approach and nested processing relationships. Additional examples for seed cotton, maize, wheat, and their products are provided in Supplementary Information (SI) Figs. S1–S3.

**Fig. 2:**
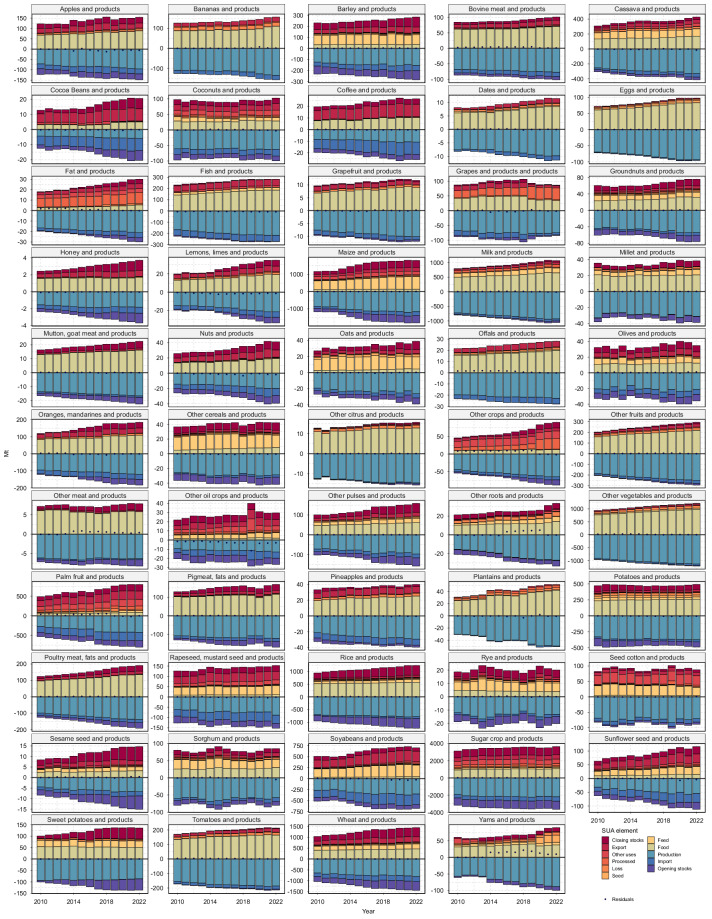
Traceable FBS (T-FBS): supply utilization balances in Primary Commodity equivalents (PCe). Regional supply (negative values, represented by filled color bars) and regional demand (positive values, represented by filled color bars) are displayed for aggregated PCe commodities from 2010 to 2022 at the world level (covering 195 regions). Gross trade values are included, with gross exports equaling gross imports annually at the world level. Residuals, representing the difference between regional supply and demand, are indicated by points. The figure shows data for 54 PCe commodities (see Table S1 for commodity mappings) while “Other fiber crops and products”, “Livestock meat equivalent”, and “NEC” (not elsewhere categorized) are excluded for simplicity, as they are not the main focus due to the absence of food consumption or their straightforward aggregation.

**Fig. 3:**
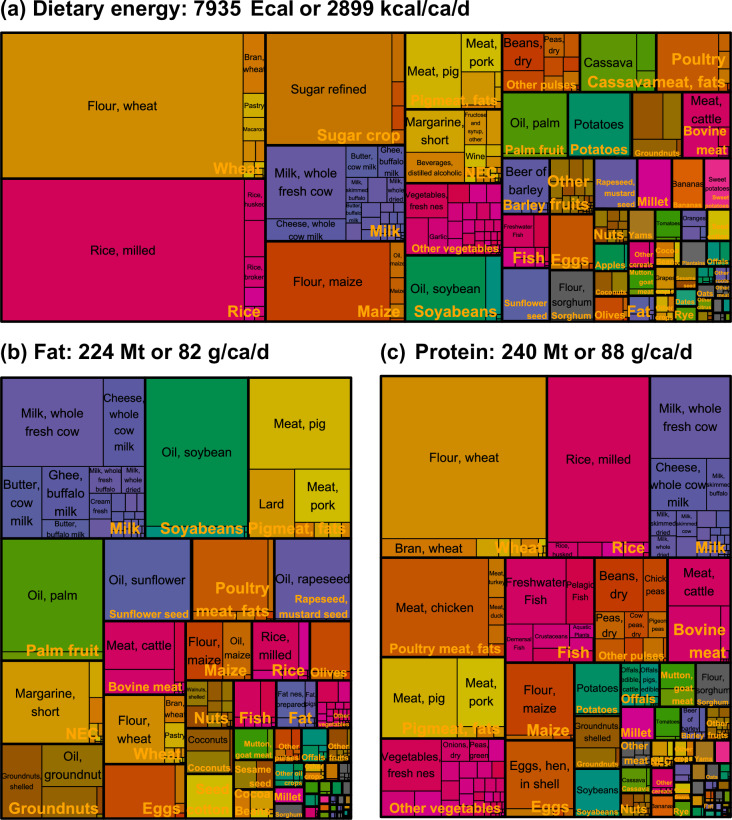
Traceable FBS (T-FBS): global dietary energy, fat, and protein supplies by aggregated PCe and SUA commodities (mean values for 2010–2022). The treemaps illustrate the distribution of food supply by aggregated PCe commodities (larger rectangles; orange labels) and their corresponding SUA commodities (smaller rectangles; black labels) for dietary energy (Panel a), fat (Panel b), and protein (Panel c). See Table S1 for commodity mappings, and the “and products” in the label of PCe commodities is omitted for simplicity. The total global (195 regions) supply per year is provided in Exa calories (Ecal) or million tonnes (Mt), while the average per capita daily supply is shown in kilocalories per capita per day (kcal/ca/d) or grams per capita per day (g/ca/d), as indicated in the panel titles.

The newly compiled T-FBS dataset includes 57 PCe commodities aggregated from over 500 SUA commodities. Figure 2 provides a global overview of the supply and utilization balances for PCe commodities, while Fig. 3 shows the corresponding global dietary energy, fat, and protein supplies (averaged over 2010–2022) by aggregated PCe commodities and the finer-sectoral-resolution SUA commodities. Notably, a residual term representing the difference between regional supply and demand may exist. While residuals in PCe in T-FBS are largely inherited from the SUA data, our study underscores the need for further efforts to enhance data quality (i.e., minimizing residuals) within a transparent and traceable framework. The approach would also benefit significantly from greater transparency in the methodology used to compile the SUA.

A comparison with the original FAO-FBS for key commodities is provided in the Technical Validation section. The resulting dataset is intended to support scientific applications such as environmental footprint analysis or economic modeling. By ensuring consistency between the FBS and SUA, the PCe approach and dataset enhance the scientific robustness of studies relying on this data for empirical analysis^20^, data development^21,22^, and the modeling of global agrifood systems and multisector dynamics^23–26^.

## Methods

### Primary commodity equivalents (pce) aggregation of supply utilization accounts (SUA)

In the SUA data, the regional supply of a commodity, *i*, in a region *r* and year *t* is calculated as the sum of the physical quantity flows, *Q*_*i,r,t,e*_, across the corresponding “accounts” or “elements” (*e*) of supply, i.e., opening stock, production, and import, as shown in Eq. (1). Similarly, regional demand is determined by summing the corresponding demand elements, as presented in Eq. (2). Note that “demand” and “utilization” are used interchangeably when describing elements or market balances in this study. The regional demand elements in Eq. (2) include those present in the SUA data used in this study. It is worth noting that these elements can be further expanded to incorporate additional categories, such as tourist consumption or bioenergy uses, if detailed in the SUA. Ideally, regional supply equals regional demand, although a residual term may account for discrepancies in data collection and processing, as indicated in Eq. (3).

1 $${{Regional\; supply}}_{i,r,t}={\sum }_{e}{Q}_{i,r,t,e},e\in \left\{{\rm{opening\; stock}},{\rm{production}},{\rm{import}}\right\}$$

2 $${{Regional\; demand}}_{i,r,t}={\sum }_{e}{Q}_{i,r,t,e},e\in \left\{{\rm{closing\; stock}},{\rm{export}},{\rm{food}},{\rm{feed}},{\rm{processed}},{\rm{seed}},{\rm{loss}},{\rm{other\; uses}}\right\}$$

3 $${{Regional\; supply}}_{i,r,t}={{Regional\; demand}}_{i,r,t}+{Q}_{i,r,t,{residuals}}$$

When fully expanded to include all supply and demand elements, Eq. (3) represents the complete supply utilization balance. In addition to the supply-demand balance identity, the SUA also includes a trade balance identity across regions, i.e., global net trade equals zero (Eq. 4) and a storage carryover identity over time (Eq. 5).

4 $${\sum }_{r}{Q}_{i,r,t,{\boldsymbol{import}}}={\sum }_{r}{Q}_{i,r,t,{\boldsymbol{export}}}$$

5 $${Q}_{i,r,t,{\boldsymbol{closing\; stock}}}={Q}_{i,r,t+1,{\boldsymbol{opening\; stock}}}$$

These identities (Eqs. 1–5) ensure balance and consistency between total supply and demand in physical units across regions and years. The same balance must be maintained during the aggregation across regions, products, and along the processing chains. Here, we present a traceable and scalable primary equivalent aggregation approach (PCe) to compile the FBS based on the SUA data.

Similar to the FAO-FBS compilation approach, we rely on extraction rates, which define the gross processing relationship between primary and processed products. However, it is important to note that the extraction rates are specific to commodity, processing flow, region, and year. Specifically, the extraction rate $E{R}_{j,k,r,t}$, for processing the primary commodity *j* into a set of secondary commodities *k* (assuming a single-level processing nest), in region *r* and year *t*, is calculated as the ratio between the total output of secondary production (${\sum }_{{\boldsymbol{k}}}{Q}_{{\boldsymbol{k}},r,t,{\boldsymbol{production}}}$) and the processed utilization of the primary commodity (${Q}_{{\boldsymbol{j}},r,t,{\boldsymbol{processed}}}$), as shown in Eq. (6).

6 $${{ER}}_{j,k,r,t}=\frac{{\sum }_{{\boldsymbol{k}}}{Q}_{{\boldsymbol{k}},r,t,{\boldsymbol{production}}}}{{Q}_{{\boldsymbol{j}},r,t,{\boldsymbol{processed}}}},k\in \left\{{\rm{secondary\; commodities\; processed\; from\; j}}\right\}$$

It is important to note that the extraction rates defined here provide a data-based gross measure of the physical flows between a primary commodity and its processed products. The underlying assumptions are: (1) the processing is complete (i.e., secondary outputs exhaust the processed use of the primary commodity), and (2) all secondary commodities share the same gross extraction rate (even though the *k* index is retained in *ER* in Eq. 6). In many cases, this approach reflects the conversion efficiency of the processing and allows for the derivation of processing losses. This relation is needed to ensure consistency between input and output mass flows after processing.

The above-mentioned assumptions are reasonable for processes with a single output or fixed coproduction relationships. However, in cases where a primary commodity is used in multiple independent processing streams to produce different outputs, and the shares of the primary commodity allocated to these processes are unknown, relying on a single gross extraction rate implied by the data may not be sufficient to represent the downstream multiple-product flows. Instead, output-specific extraction rates can be applied if additional information is available to support such distinctions. To address this, we generalize Eq. (6) to allow for user-defined output-specific extraction rates (${{ER}}_{j,k,r,t}^{{output}-{specific}}$) in combination with gross conversion coefficients (*CE*_*j,r,t*_), as presented in Eq. (7).

7 $${Q}_{{\boldsymbol{j}},r,t,{\boldsymbol{p}}{\boldsymbol{r}}{\boldsymbol{o}}{\boldsymbol{c}}{\boldsymbol{e}}{\boldsymbol{s}}{\boldsymbol{s}}{\boldsymbol{e}}{\boldsymbol{d}}}=\frac{{\sum }_{{\boldsymbol{k}}}({Q}_{{\boldsymbol{k}},r,t,{\boldsymbol{p}}{\boldsymbol{r}}{\boldsymbol{o}}{\boldsymbol{d}}{\boldsymbol{u}}{\boldsymbol{c}}{\boldsymbol{t}}{\boldsymbol{i}}{\boldsymbol{o}}{\boldsymbol{n}}}/{ER}_{j,k,r,t}^{output-specific})}{{CE}_{j,r,t}},k\in \{{\rm{s}}{\rm{e}}{\rm{c}}{\rm{o}}{\rm{n}}{\rm{d}}{\rm{a}}{\rm{r}}{\rm{y}}\,{\rm{c}}{\rm{o}}{\rm{m}}{\rm{m}}{\rm{o}}{\rm{d}}{\rm{i}}{\rm{t}}{\rm{i}}{\rm{e}}{\rm{s}}\,{\rm{p}}{\rm{r}}{\rm{o}}{\rm{c}}{\rm{e}}{\rm{s}}{\rm{s}}{\rm{e}}{\rm{d}}\,{\rm{f}}{\rm{r}}{\rm{o}}{\rm{m}}\,{\rm{j}}\}$$

The conversion coefficients are not differentiated by secondary commodities and can be derived based on known factors in Eq. (7). The updated extraction rate is calculated as the product of ${{ER}}_{j,k,r,t}^{{output}-{specific}}$ and *CE*_*j,r,t*_, as shown in Eq. (8).

8 $${{ER}}_{j,k,r,t}={{ER}}_{j,k,r,t}^{{output}-{specific}}{CE}_{j,r,t}$$

When ${{ER}}_{j,k,r,t}^{{output}-{specific}}=1$ for all secondary commodities in a processing (the default assumption), Eq. (7) collapses into Eq. (6) after rearrangement, with conversion coefficients directly representing the extraction rates (i.e., ${{ER}}_{j,k,r,t}={CE}_{j,r,t}$ for any *k*). In practice, there could also be multiple primary commodities, and additional considerations may be needed.

To convert secondary commodities (derivatives) into their PCe, all elements in the SUA (Eq. 3) of the secondary commodities are scaled using extraction rates. However, directly applying, *ER*_*j,k,r,t*_, to the supply utilization balance structure would disrupt the trade and storage balances (Eqs. 4, 5). Since *ER*_*j,k,r,t*_ represents domestic processing for a specific year, it should only be applied to the SUA elements associated with domestic production (e.g., regional supply) for that year. It is important to account for where and when the processing occurred, as the corresponding extraction rates may vary due to factors such as technological progress or differences in technology mixes. Therefore, different extraction rates should be applied to balance elements associated with opening stock and imports, depending on the source and year of the processing.

For converting the supply utilization balances of a secondary commodity *k*, *Q*_*k,r,t,e*_, into its PCe for the primary commodity *j*, ${Q}_{k,r,t,e,j}^{{PCe}}$, we first decompose *Q*_*k,r,t,e*_ by the regional supply accounts into ${Q}_{k,r,t,e}^{{imported}}$, ${Q}_{k,r,t,e}^{{domestic}:{opening\; stock}}$, and ${Q}_{k,r,t,e}^{{domestic}:{production}}$. For PCe scaling, each component is then associated with the corresponding extraction rate, ${{ER}}_{j,k,t}^{{imported}}$, ${{ER}}_{j,k,r,t-1}$, and ${{ER}}_{j,k,r,t}$, respectively, based on the source of processing, as shown in Eq. (9).

9 $${Q}_{k,r,t,e,j}^{{PCe}}=\frac{{Q}_{k,r,t,e}^{{imported}}}{{{ER}}_{j,k,t}^{{imported}}}+\frac{{Q}_{k,r,t,e}^{{domestic}:{opening\; stock}}}{{{ER}}_{j,k,r,{\boldsymbol{t}}-{\bf{1}}}}+\frac{{Q}_{k,r,t,e}^{{domestic}:{production}}}{{{ER}}_{j,k,r,t}}$$

Specifically, for processed products that are imported (${Q}_{k,r,t,e}^{{imported}}$), both the import and the corresponding utilizations are scaled using an international extraction rate (${{ER}}_{j,k,t}^{{imported}}$) (Eq. 7), which is calculated as the export-weighted average extraction rate across exporting regions for that commodity (Eq. 10). For processed products carried over from the previous period in opening stock (${Q}_{k,r,t,e}^{{domestic}:{opening\; stock}}$), a lagged extraction rate (${{ER}}_{j,k,r,{\boldsymbol{t}}-{\boldsymbol{1}}}$) is applied to both the opening stock and its utilizations. Although stocks can be carried over multiple years, this is typically not the case for agricultural products due to the first-in-first-out rule and cost considerations, as these are mostly perishable products. Finally, the domestic extraction rate in the current year (${{ER}}_{j,k,r,t}$) is applied to domestic production and its corresponding utilization (Eq. 7).

10 $${{ER}}_{j,k,t}^{{imported}}=\frac{{\sum }_{r}({Q}_{k,r,t,{\boldsymbol{export}}}\cdot {{ER}}_{j,k,r,t})}{{\sum }_{r}{Q}_{k,r,t,{\boldsymbol{export}}}}$$

By applying differentiated extraction rates, the international trade and storage balance identities, i.e., Eqs. (4, 5), are preserved in ${Q}_{k,r,t,e,j}^{{PCe}}$. Similarly, as regional demand and supply balances are demonstrated for a commodity in Eqs. (1–3), we also decompose the PCe scaling process in Eq. (9) by regional supply and utilization accounts, as shown in Eqs. (11–13). While the PCe residual term ${Q}_{k,r,t,{residuals}}^{{PCe}}$ can be traced back to residuals in the SUA of processed commodities, where applicable, it is also used to absorb any potential discrepancies introduced by the PCe approach.

11 $${{Regional\; supply}}_{k,r,t,j}^{{PCe}}=\frac{{{Regional\; supply}}_{k,r,t}^{{imported}}}{{{ER}}_{j,k,t}^{{imported}}}+\frac{{{Regional\; supply}}_{k,r,t}^{{domestic}:{opening\; stock}}}{{{ER}}_{j,k,r,{\boldsymbol{t}}-{\bf{1}}}}+\frac{{{Regional\; supply}}_{k,r,t}^{{domestic}:{production}}}{{{ER}}_{j,k,r,t}}$$

12 $${{Regional\; demand}}_{k,r,t,j}^{{PCe}}=\frac{{{Regional\; demand}}_{k,r,t}^{{imported}}}{{{ER}}_{j,k,t}^{{imported}}}+\frac{{{Regional\; demand}}_{k,r,t}^{{domestic}:{opening\; stock}}}{{{ER}}_{j,k,r,{\boldsymbol{t}}-{\bf{1}}}}+\frac{{{Regional\; demand}}_{k,r,t}^{{domestic}:{production}}}{{{ER}}_{j,k,r,t}}$$

13 $${{Regional\; supply}}_{k,r,t,j}^{{PCe}}={{Regional\; demand}}_{k,r,t,j}^{{PCe}}+{Q}_{k,r,t,{\boldsymbol{residuals}},j}^{{PCe}}$$

This ensures that the supply and demand balances remain undisrupted as long as the underlying relationships hold at the level of the decomposed regional supply terms, as illustrated in Eqs. (14–16). In practice, we assume that exports and closing stock are supplied by domestic production, and we distribute the imports and opening stocks among the rest of the utilization accounts to match the corresponding regional supply, as shown in Eqs. (17, 18). For simplicity, we apply the same share across the related utilization accounts in our assumption. While there may be some uncertainty in this allocation approach, the detailed data flows (by opening stock, imported, or domestic supply) are typically not observable from the perspective of market competition. The domestic production is then calculated as the residual (Eq. 19).

14 $${{Regional\; supply}}_{k,r,t}^{{imported}}={Q}_{k,r,t,{\boldsymbol{import}}}={{Regional\; demand}}_{k,r,t}^{{imported}}$$

15 $${{Regional\; supply}}_{k,r,t}^{{domestic}:{opening\; stock}}={Q}_{k,r,t,{\boldsymbol{opening\; stock}}}={{Regional\; demand}}_{k,r,t}^{{domestic}:{opening\; stock}}$$

16 $${{Regional\; supply}}_{k,r,t}^{{domestic}:{production}}={Q}_{k,r,t,{\boldsymbol{production}}}={{Regional\; demand}}_{k,r,t}^{{domestic}:{production}}+{Q}_{k,r,t,{residuals}}$$

17 $${{Regional\; demand}}_{k,r,t}^{{imported}}={\sum }_{e}{Q}_{k,r,t,e}^{{imported}},e\in \left\{{\rm{food}},{\rm{feed}},{\rm{processed}},{\rm{seed}},{\rm{loss}},{\rm{other\; uses}}\right\}$$

18 $$\begin{array}{l}{{Regional\; demand}}_{k,r,t}^{{domestic}:{opening\; stock}}={\sum }_{e}{Q}_{k,r,t,e}^{{domestic}:{opening\; stock}},\\ \,\,\,\,\,\,\,\,\,\,\,\,\,e\in \left\{{\rm{food}},{\rm{feed}},{\rm{processed}},{\rm{seed}},{\rm{loss}},{\rm{other\; uses}}\right\}\end{array}$$

19 $${{Regional\; demand}}_{k,r,t}^{{domestic}:{production}}=\left\{\begin{array}{l}{Q}_{i,r,t,e}-{Q}_{i,r,t,e}^{{imported}}-{Q}_{i,r,t,e}^{{domestic}:{opening\; stock}},\\ e\in \left\{{\rm{food}},{\rm{feed}},{\rm{processed}},{\rm{seed}},{\rm{loss}},{\rm{other\; uses}}\right\}\\ {Q}_{i,r,t,e},e\in \left\{{\rm{closing\; stock}},{\rm{export}}\right\}\end{array}\right.$$

Equations (1–19) ensure consistency and balance in our data processing. Once the PCe conversion of secondary commodities is complete, the final step is to aggregate the primary commodities and PCe of secondary commodities to provide an aggregated representation of the supply chain (${Q}_{r,t,e,j}^{{PCe}}$), as shown in Eq. (20). In this step, the production account of the primary product $j$ is preserved, while the processed use of the primary product is explained by the supply utilization balances of the secondary products, ensuring it is offset by the production of those secondary products. For all other balance elements, the primary and secondary products are summed together.

20 $${Q}_{r,t,e,j}^{{PCe}}=\left\{\begin{array}{l}{Q}_{j,r,t,e},e\in \left\{{\rm{production}}\right\}\\ {Q}_{j,r,t,e}-{\sum }_{k}{Q}_{k,r,t,{\boldsymbol{production}},j}^{{PCe}},e\in \left\{{\rm{processed}}\right\}\\ {Q}_{j,r,t,e}+{\sum }_{k}{Q}_{k,r,t,e,j}^{{PCe}},e\notin \left\{{\rm{production}},{\rm{processed}}\right\}\end{array}\right.$$

**Fig. 4:**
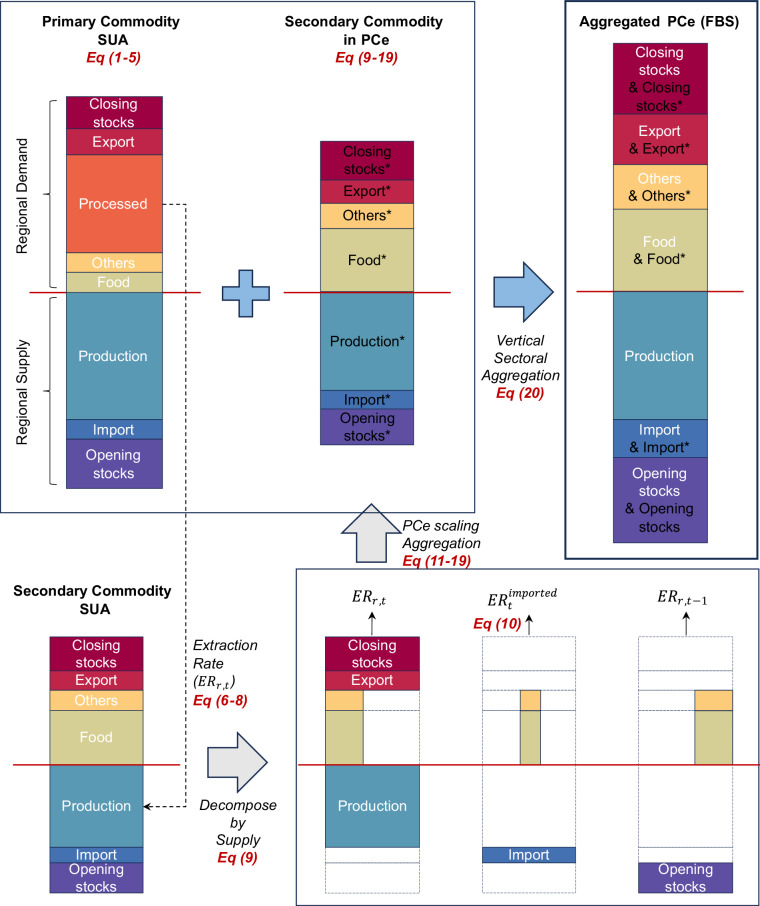
Schematic diagram of the Primary Commodity equivalents (PCe) aggregation process from Supply Utilization Accounts (SUA). The aggregated PCe, representing commodities in the Food Balance Sheets (FBS), is obtained through vertical sectoral aggregation (blue arrow) of supply utilization balances for both the primary commodity and the secondary commodity expressed in PCe. The supply utilization balances of the secondary commodity are transformed into PCe by applying differentiated extraction rates (ER), with data flows represented by gray arrows. In this diagram, regional supply (above the red horizontal divider line) and regional demand (below the red horizontal divider line) are balanced, with individual elements (filled color bars) annotated. Asterisks are added to the account names for secondary commodities in PCe to distinguish them after aggregation. Relevant equations (Eq) from the Methods section are annotated within the schematic diagram.

A schematic illustration of the PCe aggregation process and its corresponding data flow (Eqs. 1–20) is shown in Fig. 4.

### Primary commodity equivalents aggregation of dietary energy and macronutrient accounts

A key feature of the FBS is its ability to provide aggregated accounts of dietary energy (calories) and macronutrients (proteins and fats). For these accounts (e), which include calories, proteins, and fats, the values can be summed across all primary and secondary commodities involved in the primary equivalent aggregation to derive the corresponding accounts in PCe (Eq. 21).

21 $${Q}_{r,t,e,j}^{{PCe}}={Q}_{j,r,t,e}+{\sum }_{k}{Q}_{k,r,t,e},e\in \left\{{\rm{calories}},{\rm{proteins}},{\rm{fats}}\right\}$$

While dietary energy and macronutrients maintain a relatively fixed relationship with food supply measured in mass units at fine sectoral scales (primary or processed food products), this relationship is affected during the aggregation process. As a result, the calorie and macronutrient conversion rates from the food supply in mass units for the PCe in the FBS may differ from those of the primary commodity and must be recalculated after aggregation. In addition, note that while carbohydrates are not explicitly included here (consistent with FAO-FBS), they can be derived from the relationship between total dietary energy supply and the energy contributions from proteins and fats.

### Data-based scalable approach for compiling FBS

The PCe aggregation method developed in this study (Eqs. 1–21) is generalizable and scalable, enabling the generation of the T-FBS dataset based on data available in the SUA dataset. A harmonized and balanced SUA dataset, recompiled based on FAO datasets^27^, has previously been made available through *gcamfaostat*^19^. The recompiled SUA dataset harmonizes key elements from various original FAO datasets, including supply utilization accounts (with an FAOSTAT dataset code of SCL), commodity balances (CBH), food balances (FBS and FBSH), crops and livestock products (QCL), gross trade (TCL), and bilateral trade (TM); see further details in the data processing section of the *gcamfaostat* documentation. Additionally, in the utilization elements, where applicable, “tourist consumption” has been aggregated into “food” due to its limited regional and sectoral coverage.

**Fig. 5:**
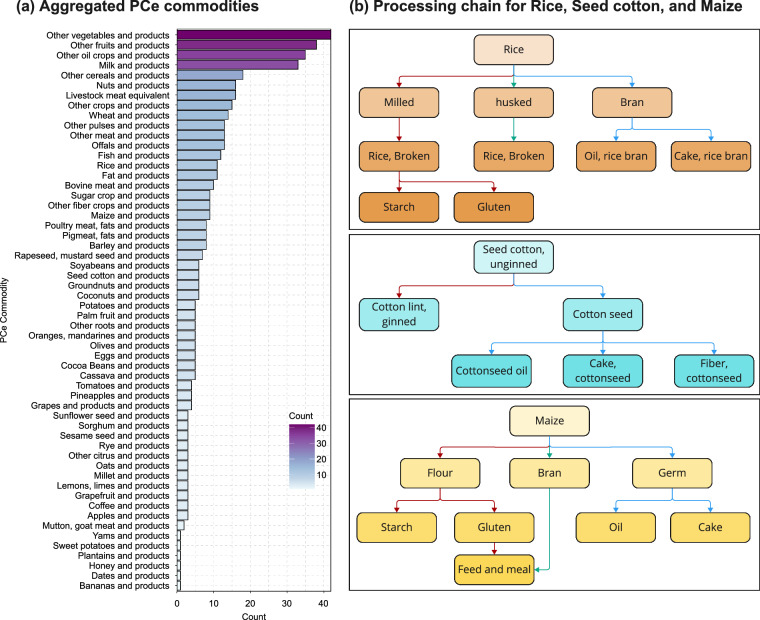
Product categories resulting from the aggregation of Primary Commodity equivalents (PCe) and underlying chain structures. Panel (**a**) presents a histogram showing the distribution of the SUA commodities (485 in total) across the 56 aggregated PCe commodities. The NEC (Not Elsewhere Categorized) group is excluded. Panel (**b**) illustrates the nesting structure of the processing chains for Rice, Seed Cotton, and Maize.

Using FAO commodity definitions and mappings^17,28^, we developed a nested mapping (SI Table S1) that aggregates over 500 commodities in the SUA dataset into 57 aggregated PCe commodities in T-FBS through backward aggregation along nest levels (processing chains). Two aggregated PCe commodities, “Livestock meat equivalent” (derived by converting live animal stocks to their meat equivalent using carcass yield) and “NEC” (Not Elsewhere Categorized), were included for completeness. Figure 5 presents the count of SUA commodities by the aggregated PCe commodities and illustrates the nesting structure of the processing chains for rice and products, seed cotton and products, and maize and products. The T-FBS dataset includes 195 regions (Table S2), consistent with the SUA dataset but exceeding the number of regions (186) included in the FAO-FBS.

Compared to the 97 commodities in the FAO-FBS, T-FBS is currently more sectorally aggregated due to the following reasons: (1) vegetable oils and cakes are aggregated into their PCe, whereas they are kept separate in the FAO-FBS; (2) fisheries and aquaculture products are aggregated into “fish and products” since the SUA data are not available and they are not the primary focus of this study; and (3) additional aggregation is applied at the PCe level (from PCe commodities to aggregated PCe commodities; see Table S1) to simplify the data and emphasize key commodities. It is worth noting that the new PCe approach is scalable, employing a hierarchical structure that allows for flexible disaggregation of processing sectors or further aggregation along the processing chain as more detailed downstream data (or improved mapping of NEC commodities) become available.

New functions and features supporting this process have been incorporated into *gcamfaostat v1.1*. All input, intermediate, and output data are fully traceable within *gcamfaostat v1.1*, facilitated by the implementation of the *drake* data pipeline system^29^. The approach is largely data-driven, as the commodity-nest_level-region-year-specific extraction rates are mainly calculated based on the processing relationships inferred from the detailed data, with potential output-specific rates applied when multiple secondary products are present. A minimum threshold value was applied in regions where computed extraction rates were unreasonably low, which could lead to excessive over-scaling of secondary commodity balances. The extraction rates used in this study may differ from those used in compiling the FAO-FBS, as the latter relies on non-publicly available data that is not accessible. To enhance transparency, the extraction rates used in this study have been made publicly available alongside the T-FBS dataset. Future research is needed to further examine and refine these extraction rates.

Although T-FBS methodology ensures balance and scalability across multiple dimensions, its accuracy is inherently dependent on the quality and completeness of the input SUA dataset. Specifically, uncertainties in extraction rates and balance adjustments for trade and stock data can impact the results for certain commodities or regions. Additionally, while the PCe approach provides a scalable framework, its implementation relies on assumptions about the relationships between primary and processed commodities. Complementary data and additional assumptions are often required to fully represent processing chains within a data-based approach. These factors highlight the importance of continuous refinement and validation of the input data and assumptions. The T-FBS is currently compiled only for 2010–2022 due to the availability of the SUA dataset. However, our approach can be directly applied to additional years once historical SUA data (pre-2010) or new data (post-2022) become available.

## Data Records

The newly compiled T-FBS is presented in two main output datasets:

1. ***Traceable_FBS_PCe_2010_2022.csv***, which contains the supply utilization balances in PCe, and
2. ***Traceable_FBS_Food_Calorie_Macronutrient_2010_2022.csv***, which details the corresponding accounts for dietary energy and macronutrients.

The main input datasets, including the recompiled SUA, and the nested mapping file linking processed commodities (sink items) to primary commodities (source items), and the extraction rates, have also been archived as supplementary data along with the T-FBS datasets. Table 1 provides a summary of these data records. All datasets were generated and maintained using *gcamfaostat v1.1* and are archived in a repository^30^ hosted on Zenodo. The data export date is included in the dataset headers and can be utilized for version tracking.

**Table 1:** Data records.

File Name	Description	Element and Unit
Traceable_FBS_PCe_2010_2022.csv	Supply utilization balances for 195 regions (ISO codes) and 57 items (aggregated primary commodity equivalents) from 2010 to 2022. This constitutes the first part of T-FBS.	15 elements: Opening stocks, Production, Import, Export, Processed, Food, Feed, Seed, Other uses, Loss, Closing stocks, Residuals, Regional supply, Regional demand, and Stock variation. All elements are measured in thousand tonnes.
SUA_2010_2022.csv	Supply utilization balances for 195 regions (ISO codes) and 529 items (SUA commodities) from 2010 to 2022. Includes FAO item codes. This dataset serves as input for compiling T-FBS.	
Traceable_FBS_Food_Calorie_Macronutrient_2010_2022.csv	Dietary energy and macronutrients (fat and protein) for 195 regions (ISO codes) and 55 food items (aggregated primary commodity equivalents) from 2010 to 2022. Two aggregated PCe items (“Livestock meat equivalent” and “Other fiber crops and products”) are excluded since they do not have food utilization. This constitutes the second part of T-FBS.	3 elements: Dietary energy, measured in million kilocalories, and Fat and Protein, measured in million tonnes.
SUA_Food_Calorie_Macronutrient_2010_2022.csv	Dietary energy and macronutrients (fat and protein) for 195 regions (ISO codes) and 431 food items from 2010 to 2022. FAO item codes are included. This dataset serves as input for compiling T-FBS.	
Nested_Mapping_SUA_To_Traceable_FBS.csv	Nested mapping between processed commodities (sink items) and primary commodities (source items) by aggregated PCe commodities (57 items) and nest levels. FAO item codes are provided for source and sink items.	Not applicable.
Traceable_FBS_Extraction_Rate_2010_2022.csv	Extraction rate by regions (ISO), year, processing (PCe commodity, source items, sink items, and nest levels) by point of processing (domestic, imported, and lagged).	Not applicable.

## Technical Validation

The data preparation, synthesis, and processing for this study were fully integrated into the open-source *gcamfaostat v1.1* package, which includes new functions and transparent data-tracing features^19^. A global overview of T-FBS is provided in Figs. 2, 3. In this section, we check data balances, examine residual terms in the supply utilization balances, and compare T-FBS with the FAO-FBS in key areas.

**Fig. 6:**
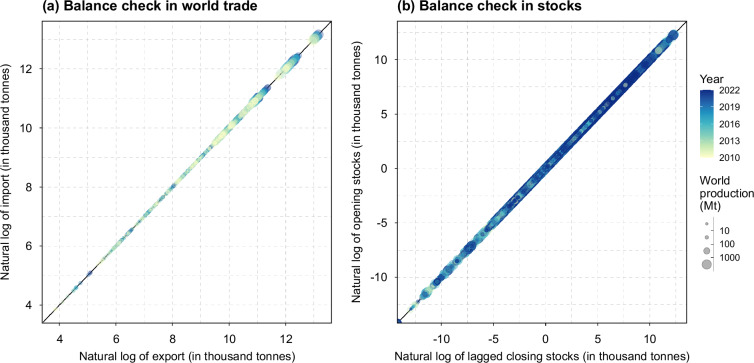
Balance check for world trade and stocks in T-FBS. Panel (**a**) compares world imports to world exports (in natural logarithmic values) for aggregated PCe commodities across years. Panel (**b**) compares opening stocks in a given year to closing stocks from the previous year (in natural logarithmic values) for aggregated PCe commodities and regions across years (excluding 2010). In both panels, point size represents the global production of the commodity (in million tonnes), and color denotes the year. A 45-degree reference line is included to indicate perfect balance between values in the x-axis and y-axis.

The accounts are balanced in world trade and stock carryover (Fig. 6), supported by balance-check and negative control features incorporated into the processing. Regional supply and demand are balanced when residuals are included as part of regional demand. Minimal adjustments were made to the residuals in the PCe aggregation process, leaving them primarily inherited from the source data. The residual account in the SUA data captures discrepancies arising from raw data collection, estimation errors, and inconsistencies across data sources^11^. However, residuals in processed commodities can be scaled and accumulated through the PCe aggregation process, making them potentially more pronounced.

**Fig. 7:**
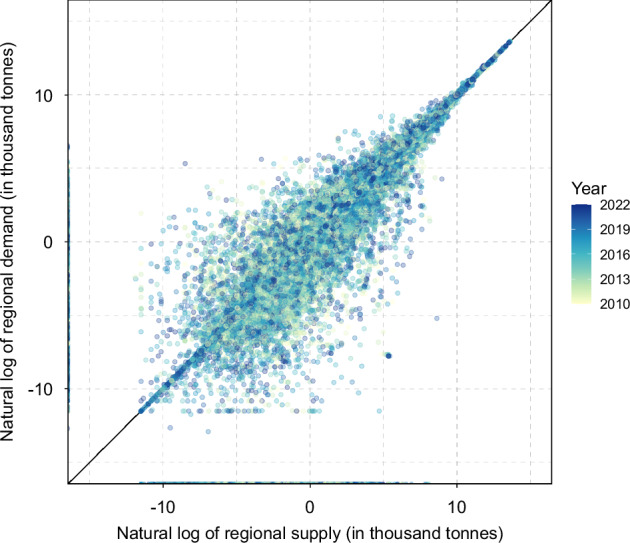
Balance check for regional supply and demand. Each point compares the total regional demand (y-axis) and total regional supply (x-axis), both measured in thousand tonnes and presented in natural logarithmic values, for each PCe commodity, country, and year (indicated by colors). Values smaller than −20 are truncated. A 45-degree reference line is included to indicate perfect agreement.

While residuals remain relatively small at the global aggregated PCe level (Fig. 2), they are not negligible and can be more pronounced at regional and commodity levels (Fig. 7), particularly for regions and commodities with smaller contributions to global production and consumption (SI Figs. S4, S5). Table 2 provides descriptive statistics of residuals pooled across regions and years for each PCe commodity. The mean residual values range from -62 thousand tonnes (kt) for NEC to 125 kt for palm fruits and products. The mean absolute error (MAE) spans from 1 kt (honey and products) to 716 kt (sugar crop and products), while the standard deviation ranges from 3 kt (honey and products) to 2,291 kt (palm fruits and products). Results from t-tests show that the mean residual is not significantly different from zero at the 5% confidence level for 27 commodities. Additionally, the Symmetry Index, which measures the absolute difference between the mean and median residuals scaled by the standard deviation, is smaller than 0.1 for 48 commodities, indicating a roughly symmetric residual distribution. A narrower and more symmetric residual distribution centered around zero suggests relatively higher data quality, as data collection and processing errors are less likely to exhibit systematic bias.

**Table 2:** Summary statistics of residuals by PCe commodities.

Commodity	Count	5th	25th	50^th^ (median)	75th	95th	Mean	p-value (t-test)	MAE	Symmetry Index
Coconuts	2523	−63	−1	0	1	40	0	1.00	30	0.00
Sorghum	2159	−20	0	0	0	11	0	0.91	14	0.00
Grapefruit	2446	−8	0	0	0	6	0	0.75	4	0.01
Other citrus	2459	−4	0	0	0	4	0	0.62	2	0.01
Oranges, mandarines	2525	−54	−2	0	6	98	−2	0.60	45	0.01
Eggs	2535	−13	0	0	1	16	0	0.50	8	0.01
Sweet potatoes	2382	−4	0	0	0	4	0	0.50	3	0.01
Sesame seed	2440	−6	0	0	0	6	1	0.43	5	0.02
Seed cotton	2400	−25	0	0	0	13	−1	0.42	12	0.02
Other pulses	2521	−57	−2	0	3	66	1	0.36	24	0.02
Sunflower seed	2480	−71	−1	0	2	72	−4	0.26	34	0.02
Pineapples	2517	−18	−1	0	1	24	−1	0.25	11	0.02
Dates	2391	−2	0	0	0	3	0	0.25	2	0.02
Coffee	2528	−32	−2	0	1	18	−1	0.23	12	0.02
Wheat	2518	−299	−31	1	36	349	11	0.20	145	0.02
Plantains	2084	−24	−1	0	0	9	−2	0.24	12	0.03
Mutton, goat meat	2528	−3	0	0	0	5	0	0.19	3	0.03
Barley	2516	−98	−3	0	4	107	7	0.19	57	0.03
Bananas	2500	−71	−1	0	2	96	5	0.16	39	0.03
Grapes	2504	−44	0	0	2	39	−5	0.15	31	0.03
Cassava	2399	−36	0	0	1	32	−18	0.13	90	0.03
Millet	2254	−4	0	0	0	4	2	0.12	3	0.03
Rapeseed, mustard seed	2492	−60	0	0	1	70	−4	0.09	28	0.03
Apples	2512	−32	−1	0	5	75	−20	0.09	67	0.03
Olives	2503	−15	−1	0	0	10	2	0.08	10	0.03
Maize	2521	−283	−11	0	14	366	17	0.08	139	0.03
Other roots	2458	−10	0	0	0	5	7	0.07	13	0.04
Soyabeans	2515	−269	−11	0	10	247	−37	0.04	139	0.04
Groundnuts	2509	−14	0	0	1	19	2	0.03	11	0.04
Cocoa Beans	2517	−16	0	0	1	10	−1	0.02	6	0.05
Sugar crop	2531	−1234	−95	3	244	2280	102	0.01	716	0.05
Oats	2453	−12	0	0	0	6	−1	0.01	5	0.05
Palm fruit	2491	−824	−30	0	15	750	125	0.01	388	0.05
Milk	2528	−252	−8	2	28	374	33	0.00	145	0.06
Rye	2023	−4	0	0	0	5	1	0.00	3	0.06
Other vegetables	2531	−167	−11	0	10	252	78	0.00	140	0.07
Honey	2489	−2	0	0	0	1	0	0.00	1	0.07
Yams	1815	−2	0	0	0	2	73	0.00	75	0.07
Nuts	2514	−22	−1	0	1	24	−5	0.00	14	0.07
Other meat	2515	−5	0	0	0	8	2	0.00	4	0.07
Lemons, limes	2477	−15	0	0	1	14	−7	0.00	15	0.08
Other oil crops	2506	−96	−3	0	1	33	−16	0.00	33	0.08
Poultry meat, fats	2535	−21	0	0	8	67	7	0.00	22	0.08
Other cereals	2509	−27	−2	0	0	12	−2	0.00	8	0.09
Pigmeat, fats	2533	−17	0	0	2	38	5	0.00	13	0.09
NEC	2531	−371	−20	0	9	140	−62	0.00	122	0.09
Other fruits	2535	−72	−4	0	6	145	13	0.00	44	0.09
Rice	2527	−252	−4	1	26	355	35	0.00	115	0.10
Other crops	2530	−14	−1	0	1	45	35	0.00	42	0.12
Fat	2535	−16	0	0	3	32	4	0.00	10	0.12
Bovine meat	2530	−14	0	0	5	69	16	0.00	25	0.12
Tomatoes	2526	−23	−1	0	5	82	12	0.00	26	0.12
Offals	2535	−24	0	0	2	48	5	0.00	14	0.12
Other fiber crops	2300	−6	0	0	0	0	−2	0.00	2	0.14
Potatoes	2514	−103	−7	0	2	40	−18	0.00	37	0.14
Livestock meat equivalent	2510	−141	−12	−1	0	0	−42	0.00	42	0.14
Fish	2377	−162	−23	−4	0	1	−30	0.00	33	0.31

This table provides descriptive statistics of residuals (difference between regional supply and demand) across regions and years, by PCe commodities, including the count of observations, percentiles (5th, 25th, 50th/median, 75th, and 95th) values, mean values, and p-values from a t-test assessing whether the mean residual is significantly different from zero. Additionally, the table includes the mean absolute error (MAE) and the Symmetry Index. Commodities are sorted by Symmetry Index to illustrate the degree of asymmetry in residuals across PCe commodities. Residuals are measured in thousand tonnes (kt). The “and products” in the label of PCe commodities is omitted. The maximum number of observations per commodity is 2535 (195 regions by 13 years), while data may not be available for all years and all regions.

**Fig. 8:**
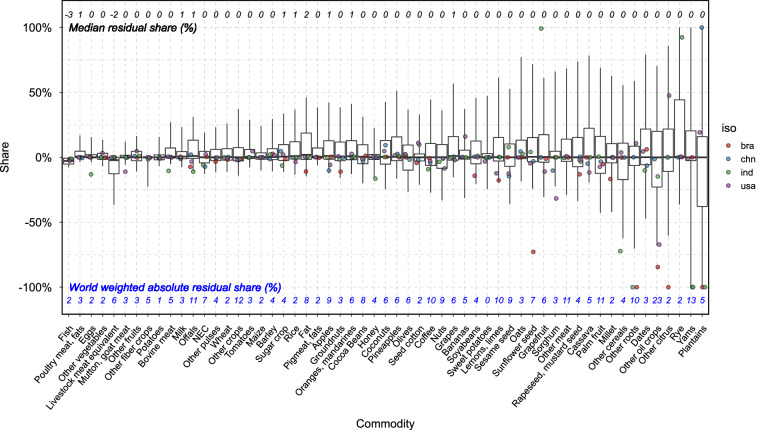
Distribution of residual share by PCe commodity. The figure displays the distribution of residual shares (%) pooled across regions and years, by PCe commodities in the x-axis. Each boxplot represents the spread of residual shares, where the central line indicates the median residual share (50th percentile), boxes mark the 25^th^ and 75^th^ percentiles, and the whiskers extend to 10^th^ to 90^th^ percentiles. Points represent residual shares for selected regions: bra (Brazil), chn (China, mainland), ind (India), and usa (the United States). The values (black) annotated above the boxplots indicate the median values for each commodity, while the blue values below represent the world aggregated (i.e., weighted by regional supply or demand) absolute residual share (%).

**Fig. 9:**
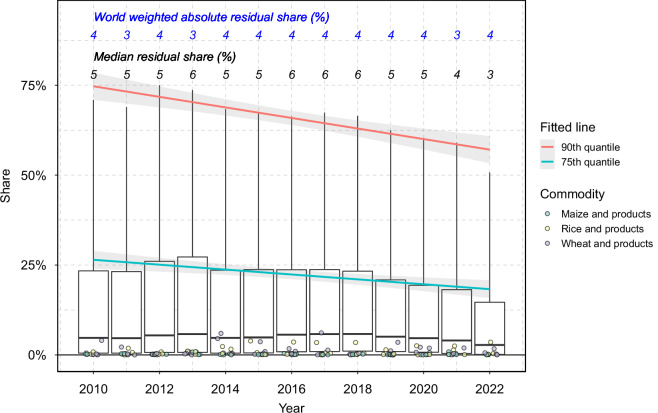
Distribution of absolute residual share across years. This figure displays the distribution of absolute residual shares (%) pooled across commodities and regions by year. Each boxplot represents the spread of absolute residual shares, with the central line indicating the median residual share (50th percentile), the boxes marking the 25th and 75th percentiles, and the whiskers extending to the 10th and 90th percentiles. The black numbers above each boxplot represent the median residual share (%), while the blue numbers below each boxplot denote the world-weighted absolute residual share (%). Colored fitted lines indicate trends for the 90th quantile (red) and 75th quantile (blue), with the ribbons representing 95% confidence intervals. Points indicate residual shares for selected commodities (maize and products, rice and products, and wheat and products) in four regions: Brazil, China (mainland), India, and the United States.

We also evaluate residuals in relative terms by computing residual shares, defined as the ratio of the residual to the maximum value between regional supply and demand. Residual shares range between −100% and 100%, providing a consistent metric for comparing data quality across commodities (Fig. 8) and years (Fig. 9). In either dimension, while the median residual shares are generally small, the variations can be substantial. Data quality also varies across commodities, with the world-aggregated absolute residual share ranging from 0% (e.g., sweet potatoes and products) to 23% (e.g., other oil crops and products). However, there is a clear trend of decreasing residual shares over time, suggesting improvements in data quality, particularly at the upper end of the residual distribution (Fig. 9). Fitted results based on 2010–2022 data show that the absolute residual shares are decreasing by 1.5 percentage points per year at the 90^th^ percentile level and 0.7 percentage points per year at the 75^th^ percentile level, with both coefficients statistically significant at the 1% confidence level.

**Fig. 10:**
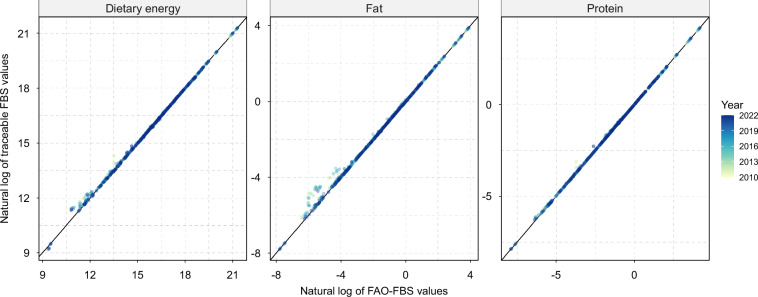
Comparison of T-FBS and FAO-FBS for dietary energy, fat, and protein supplies (2010–2022). Each point represents a comparison of dietary energy (in million kilocalories) or macronutrient supplies (fat and protein, in million tonnes) between T-FBS (y-axis) and FAO-FBS (x-axis) per country per year (indicated by color). Natural logarithmic values are used for both axes. See Table S3 for summary statistics. A 45-degree reference line is added to indicate perfect agreement.

During 2010–2022, the global supply provided an average of 2,899 kilocalories per capita per day (kcal/ca/d) of dietary energy, 82 grams per capita per day (g/ca/d) of fat, and 88 grams per capita per day (g/ca/d) of protein (Fig. 3). Figure 10 compares dietary energy, fat, and protein supplies between T-FBS and FAO-FBS at the regional level (aggregating all commodities), showing a fairly well alignment. The summary statistics is provided in SI Fig. S3. Differences between T-FBS and FAO-FBS at the world aggregated level across the years range from −18 to −3 Exa calories (Ecal) for dietary energy, −0.77 to 0.16 Mt for fat, and −0.56 to −0.12 Mt for protein. The corresponding relative differences are small, ranging from −0.4% to 0.1% for all elements. Overall, T-FBS demonstrates strong consistency with the FAO-FBS regarding dietary energy and macronutrients at the aggregated commodity levels, although differences may be more pronounced at finer sectoral levels.

Table 3 presents a comparison between T-FBS and FAO-FBS for rice and products in 2022 at the world level. The production of rice in PCe (776 Mt globally) matches the production of the primary product (rice in SUA commodities). The production of primary products is sourced from an FAO dataset (Crops and Livestock Products) that maintains the relationship with harvested area or animal numbers (e.g., crop yield or carcass yield). The relationship is not disrupted by the PCe approach. This alignment is generally true for FAO-FBS as well, although minor discrepancies may exist, likely due to differences in conversion efficiencies and assumed processing structures.

**Table 3:** Comparison between T-FBS and FAO-FBS for rice and products in 2022 at the world level.

Element	Unit	Rice and products (PCe)			SUA commodity										
		FAO-FBS	T-FBS	% diff.	Rice	Husked rice	Rice, milled	Rice, broken	Bran of rice	Rice, milled (husked)	Flour of rice	Starch of rice	Rice, gluten	Oil of rice bran	Cake of rice bran
Production	Mt	775	776	0%	776	20	435	32	55	8	2	1	0.1	1	7
Import	Mt	95	78	−18%	3	4	43	9	0	2	0.2	0.2	0	0	0
Opening stocks	Mt		383		52	5	223	13	0	4	0	0	0	1	0
**Regional supply**	**Mt**	**870**	**1237**		**831**	**29**	**701**	**55**	**55**	**14**	**2**	**2**	**0.1**	**2**	**7**
Food	Mt	638	576	−10%	2	9	398	6	4	8	2	0	0	0.5	0
Feed	Mt	56	119	112%	29	1	0.3	16	43	0.1	0	0	0	0	7
Seed	Mt	19	19	0%	19	0	0	0	0	0	0	0	0	0	0
Loss	Mt	33	33	0%	33	0	0	0	0	0	0	0	0	0	0
Processed	Mt	12	8	−34%	681	9	33	10	9	1	0	0.3	0	0.2	0
Other uses	Mt	19	20	3%	17	0	0.4	0	0	0	0	1	0.1	0.4	0
Export	Mt	88	78	−12%	3	4	43	9	0	2	0.2	0.2	0	0	0
Closing stocks	Mt		384		49	6	223	15	0	4	0	0	0	1	0
**Regional demand**	**Mt**	**866**	**1237**		**832**	**29**	**697**	**56**	**55**	**14**	**2**	**2**	**0.1**	**2**	**7**
Residuals	Mt	2	0		−1	−0.1	3	−2	0	−1	0	−0.1	0	−0.1	0
Stock variation	Mt	1.1	0.9	−19%	−3	1	0.1	2	0	1	0	0	0	0.2	0
Net export	Mt	−7	0		0	0	0	0	0	0	0	0	0	0	0
Dietary energy	Billion kcal	1492697	1502754	1%	5438	31176	1391635	21865	14938	27217	5865	157	14	4448	0
Fat	1000 t	4725	5253	11%	33	222	3580	57	775	70	22	0	0	494	0
Protein	1000 t	30547	30679	0.4%	125	702	28242	440	509	554	103	0	4	0	0

The table includes data for SUA commodities used for compiling T-FBS. Since FAO-FBS does not provide opening and closing stocks, these elements are excluded from regional supply and regional demand calculations, and thus, the residuals calculation is adjusted to account for stock variation. Values are rounded to the nearest integer or to one decimal place for values close to zero.

T-FBS offers broader regional coverage and ensures that international trade is balanced, with world net export (export minus import) equalling zero. In contrast, trade in FAO-FBS is not balanced, and values in T-FBS are typically smaller, e.g., −18% for world gross import and −12% for world gross export. While FAO-FBS provides stock variation data, it does not include opening and closing stocks. T-FBS incorporates both opening and closing stocks, making stock variation implicit, with a difference of −19% compared to FAO-FBS. Key differences between the two datasets are also observed in food (−10%), feed (+112%), and processed (−34%) utilizations for rice and products in 2022. These variations likely result from differences in processing assumptions and sectoral coverage. For example, rice bran oil and cake are included in T-FBS but not in FAO-FBS.

Adjustments in FAO-FBS are made to “proportionally spread the imbalances out among all the components” based on certain balancing mechanisms to minimize residuals. At the world level, the residual term for rice and products is small in T-FBS (0 Mt vs. 2 Mt in FAO-FBS). However, residuals can be larger at regional levels in T-FBS since they are primarily carried over from SUA commodities and no explicit adjustments are made. For instance, in India, residuals in 2022 were 0.5 Mt in T-FBS compared to 0 Mt in FAO-FBS (Table S4). For dietary energy, fat, and protein supply, differences are observed (e.g., +11% for fat compared to FAO-FBS), while they are largely explained by the inclusion of rice bran oil in T-FBS.

Similar findings emerge when comparing the two FBS datasets for maize and products (Table S5) and wheat and products (Table S6). The goal of these comparisons is to highlight key differences for transparency purposes, rather than fully explain them, while emphasizing that T-FBS provides traceability to finer sectoral scales and raw source data. Future efforts should focus on understanding the underlying assumptions of FAO-FBS and expanding the comparison to other areas.

## Usage Notes

The T-FBS (traceable Food Balance Sheets) dataset compiled in this study covers the years 2010–2022. Future updates, whether to improve existing data processing or to extend the dataset to include additional years, should be straightforward, as the code and functions for the traceable compilation of T-FBS have been made available in *gcamfaostat v1.1*. Users are encouraged to contribute to the data processing efforts within the *gcamfaostat* framework^19^.

The FBS dataset compiled by the FAO has been widely used in research on agricultural production, international trade, food availability, waste and losses, dietary health, and environmental assessments. Given its importance and widespread applications, the FBS warrants rigorous scrutiny. Our dataset, an improved version of the FBS compiled with an alternative method with enhanced traceability and scalability, can be similarly utilized in these areas of study.

The newly compiled data, T-FBS, also offers several advantages over FAO-FBS: (1) it includes additional data elements, such as opening and closing stocks; (2) it incorporates broader commodity coverage in processing, accounting for non-food commodities (e.g., oilseed cake, fiber crops, rubber); (3) the data processing framework is flexible and scalable, allowing for the inclusion of processed commodities along the partial or full processing chain; (4) it provides a more detailed interpretation of dietary energy and macronutrients due to its traceability to SUA commodities; and (5) the approach is fully transparent, verifiable, and replicable. This dataset can be used to explore additional areas of research as well as to introduce additional elements, such as tracing micronutrients.

## Supplementary information

Supplementary Information

## Data Availability

The open-source R package for preparing, processing, and synthesizing FAOSTAT data is available at github.com/jgcri/gcamfaostat/releases/tag/v1.1.0-gamma. The code for processing data and generating figures used for analysis in this study is available at github.com/realxinzhao/paper_SciData2025_tFBS_DisplayItem.
