# Supplementary material for: Traceable and Scalable Food Balance Sheets from Agricultural Commodity Supply and Utilization Accounts (2010–2022)
Source: Sci Data. 2025 May 21;12:834. doi: 10.1038/s41597-025-05137-y (PMC12095477; doi:10.1038/s41597-025-05137-y)
Supplement: Supplementary file 1 — Supplementary Information [file 41597_2025_5137_MOESM1_ESM.pdf]

Traceable and Scalable Food Balance Sheets from Agricultural Commodity Supply and Utilization Accounts

Supplementary Information

Authors

Xin Zhao[1,2], Maksym Chepeliev[3], Neus Escobar[4,5], Matthew T. Binsted[1,2], Pralit Patel[1], Page Kyle[1], Marshall A. Wise[1]

Affiliations

1. Joint Global Change Research Institute, Pacific Northwest National Laboratory, 5825 University Research Ct, College Park, MD 20740, USA

2. Center for Global Sustainability, School of Public Policy, Thurgood Marshall Hall, University of Maryland, College Park, MD 20740, USA

3. Center for Global Trade Analysis, Department of Agricultural Economics, Purdue University, 403 Mitch Daniels Blvd, West Lafayette, IN 47906, USA

4. Basque Centre for Climate Change (BC3), Scientific Campus of the University of the Basque Country, Leioa, Spain

5. Biodiversity and Natural Resources (BNR) Program, International Institute for Applied Systems Analysis (IIASA), Schlossplatz 1, 2361 Laxenburg, Austria

Corresponding author: Xin Zhao (xin.zhao@pnnl.gov)

List of Supplementary Figures and Tables

Supplementary Fig. S1 | Global supply utilization balances for seed cotton and products in primary commodity equivalents (PCe) from traceable FBS (T-FBS) and for corresponding primary and processed products from the SUA. 1
Supplementary Fig. S2 | Global supply utilization balances for maize and products in primary commodity equivalents (PCe) from traceable FBS (T-FBS) and for corresponding primary and processed products from the SUA from 2010 to 2022. 2
Supplementary Fig. S3 | Global supply utilization balances for wheat and products in primary commodity equivalents (PCe) from traceable FBS (T-FBS) and for corresponding primary and processed products from the SUA from 2010 to 2022. 3
Supplementary Fig. S4 | Balance check for regional supply and demand in the T-FBS by aggregated PCe commodities. 4
Supplementary Fig. S5 | Balance check for regional supply and demand in the T-FBS by countries (ISO). 5

Supplementary Table S1 | Mapping of items in the detailed Supply Utilization Account (SUA) dataset to aggregated Primary Commodity equivalent (PCe) commodities in the new traceable Food Balance Sheets (T-FBS). 6
Supplementary Table S2 | Regions in the new traceable Food Balance Sheets (FBS). 17
Supplementary Table S3 | Comparison of T-FBS and FAO-FBS for dietary energy, fat, and protein supplies (2010–2022). 20
Supplementary Table S4 | Comparison between T-FBS and FAO-FBS for rice and products in 2022 in India. 21
Supplementary Table S5 | Comparison between T-FBS and FAO-FBS for maize and products in 2022 at the world level. 22
Supplementary Table S6 | Comparison between T-FBS and FAO-FBS for wheat and products in 2022 at the world level. 23

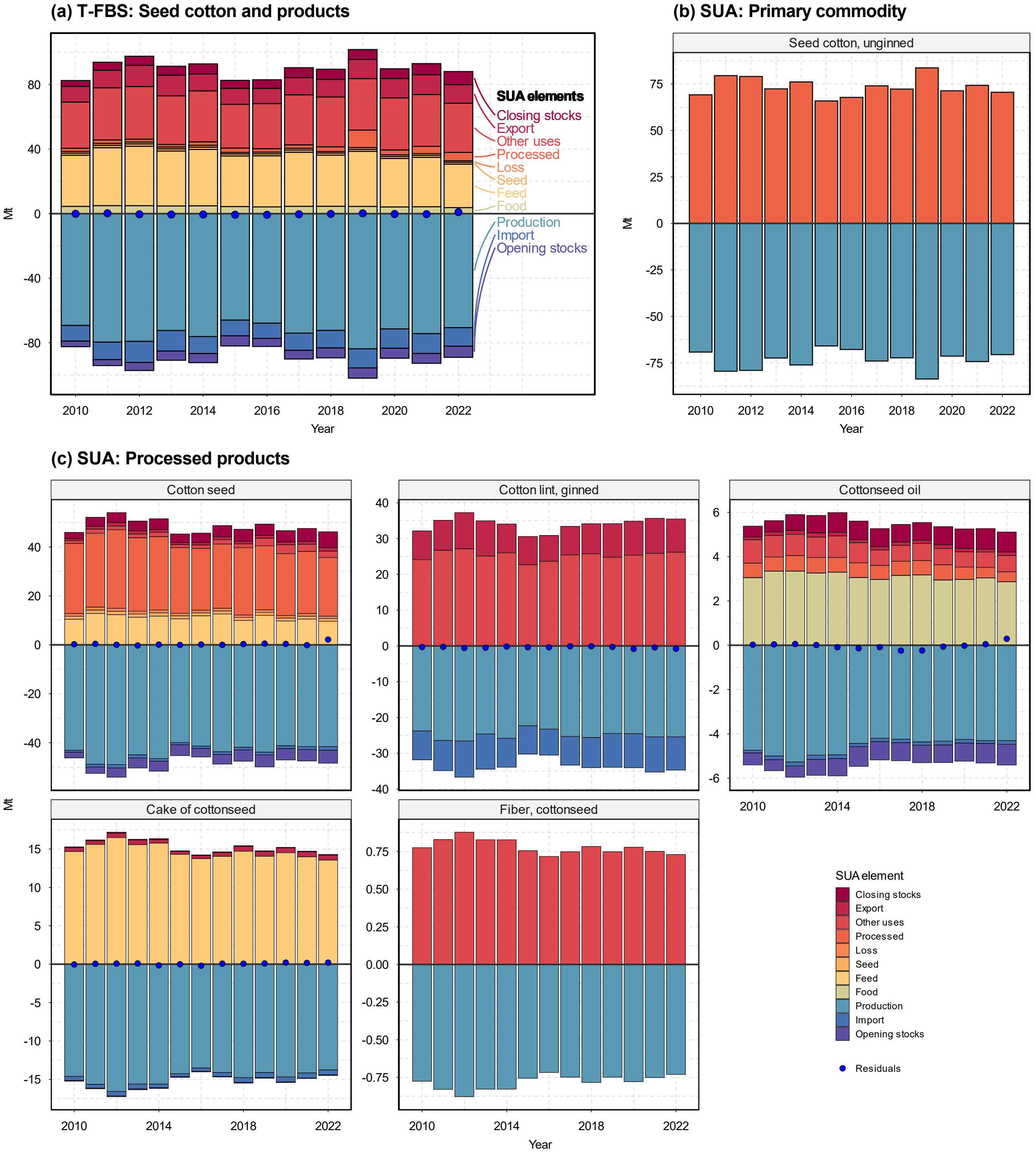

Supplementary Fig. S1 | Global supply utilization balances for seed cotton and products in primary commodity equivalents (PCe) from traceable FBS (T-FBS) and for corresponding primary and processed products from the SUA. Panel (a) illustrates the supply utilization balances in PCe from the T-FBS, while Panels (b) and (c) show the balances for the corresponding primary product (seed cotton, unginned) and processed products, respectively. Regional supply (negative values, shown as filled color bars) and regional demand (positive values, shown as filled color bars) are displayed for all commodities from 2010 to 2022 at the world level (covering 195 regions). Gross trade values are included, with gross exports equaling gross imports annually at the world level. Residuals, representing the difference between regional supply and demand, are indicated by points (not shown when Residuals are zero).

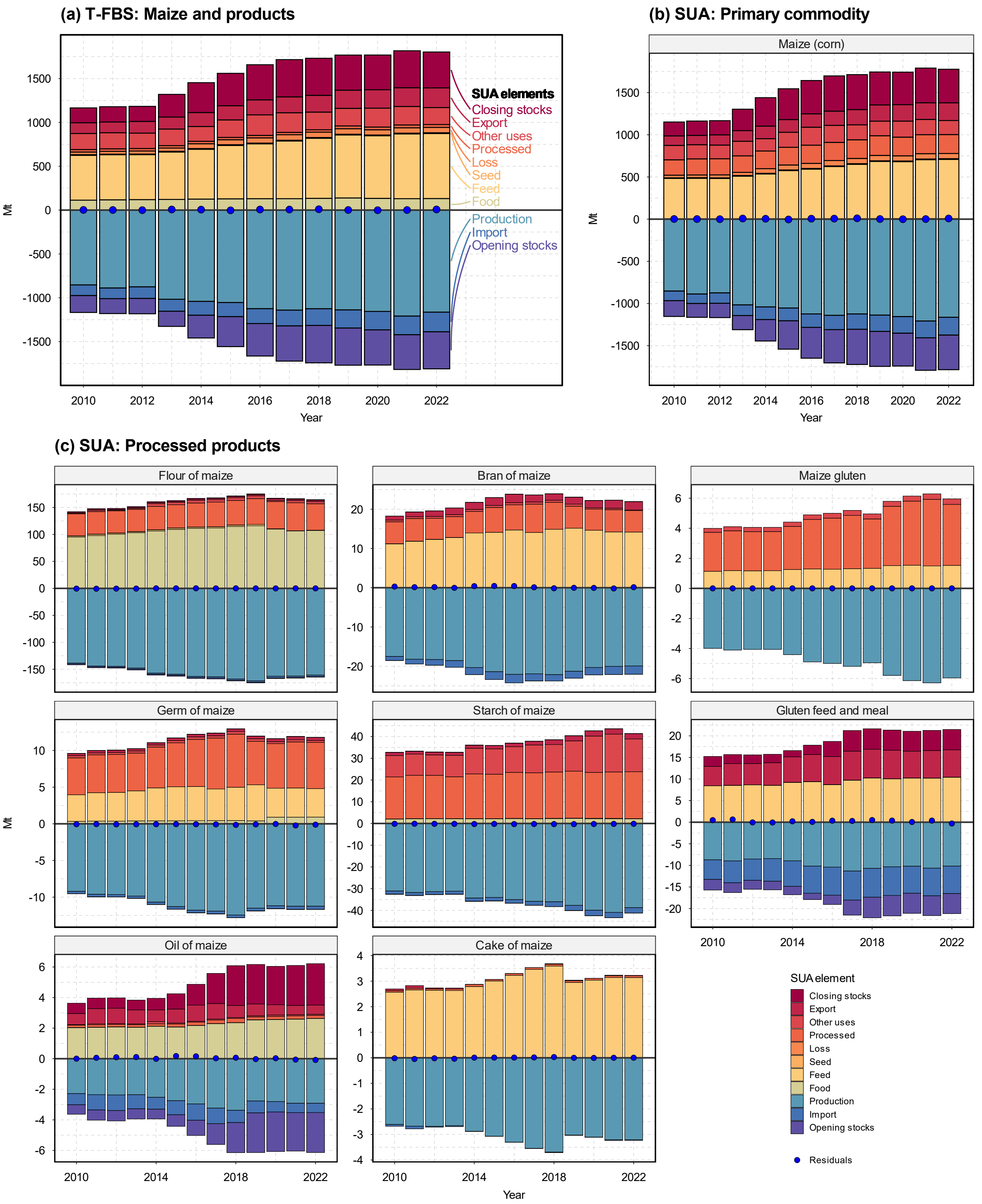

Supplementary Fig. S2 | Global supply utilization balances for maize and products in primary commodity equivalents (PCe) from traceable FBS (T-FBS) and for corresponding primary and processed products from the SUA from 2010 to 2022. See the detailed figure description in the caption of Supplementary Fig. S1.

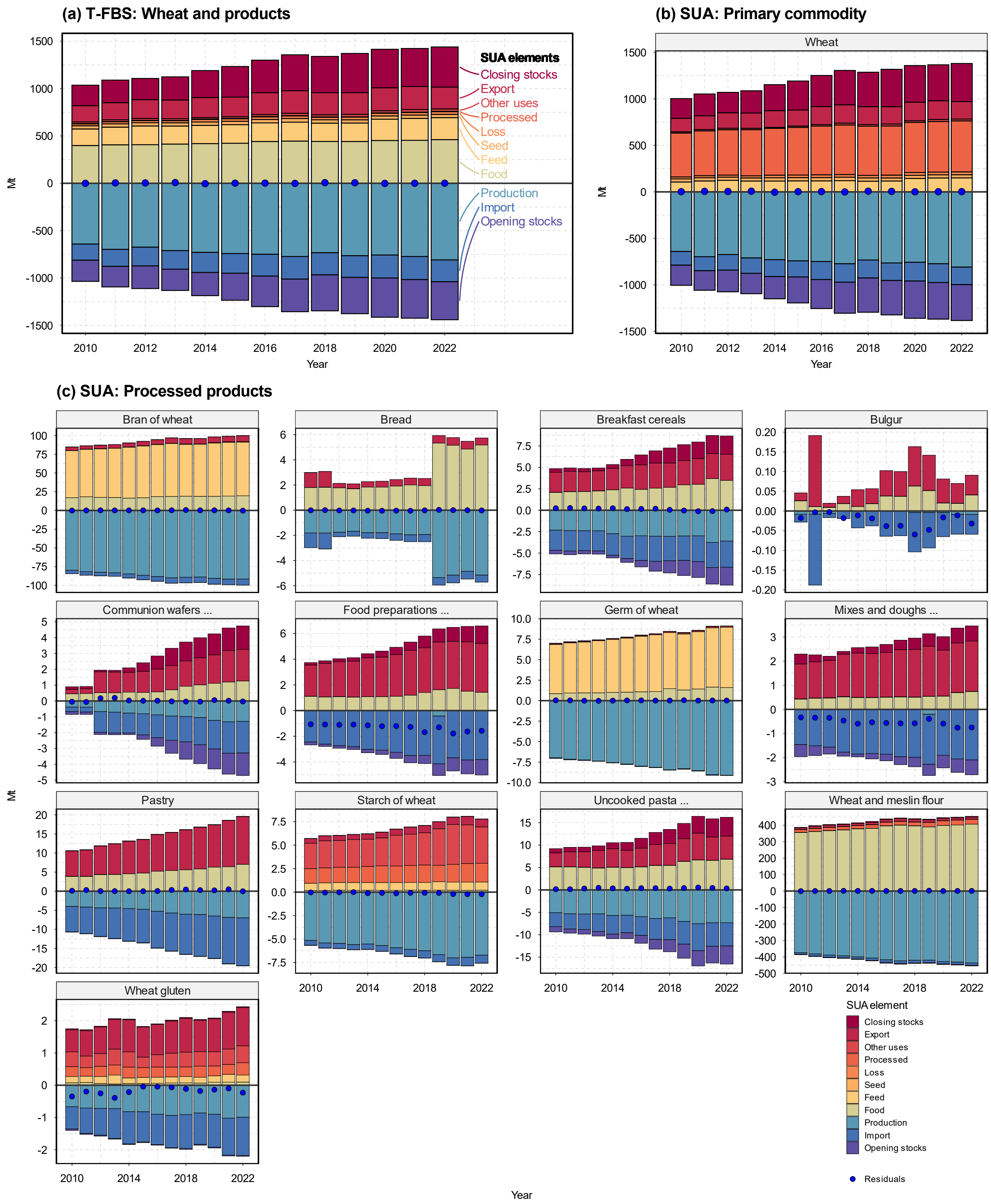

Supplementary Fig. S3 | Global supply utilization balances for wheat and products in primary commodity equivalents (PCe) from traceable FBS (T-FBS) and for corresponding primary and processed products from the SUA from 2010 to 2022. See the detailed figure description in the caption of Supplementary Fig. S1.

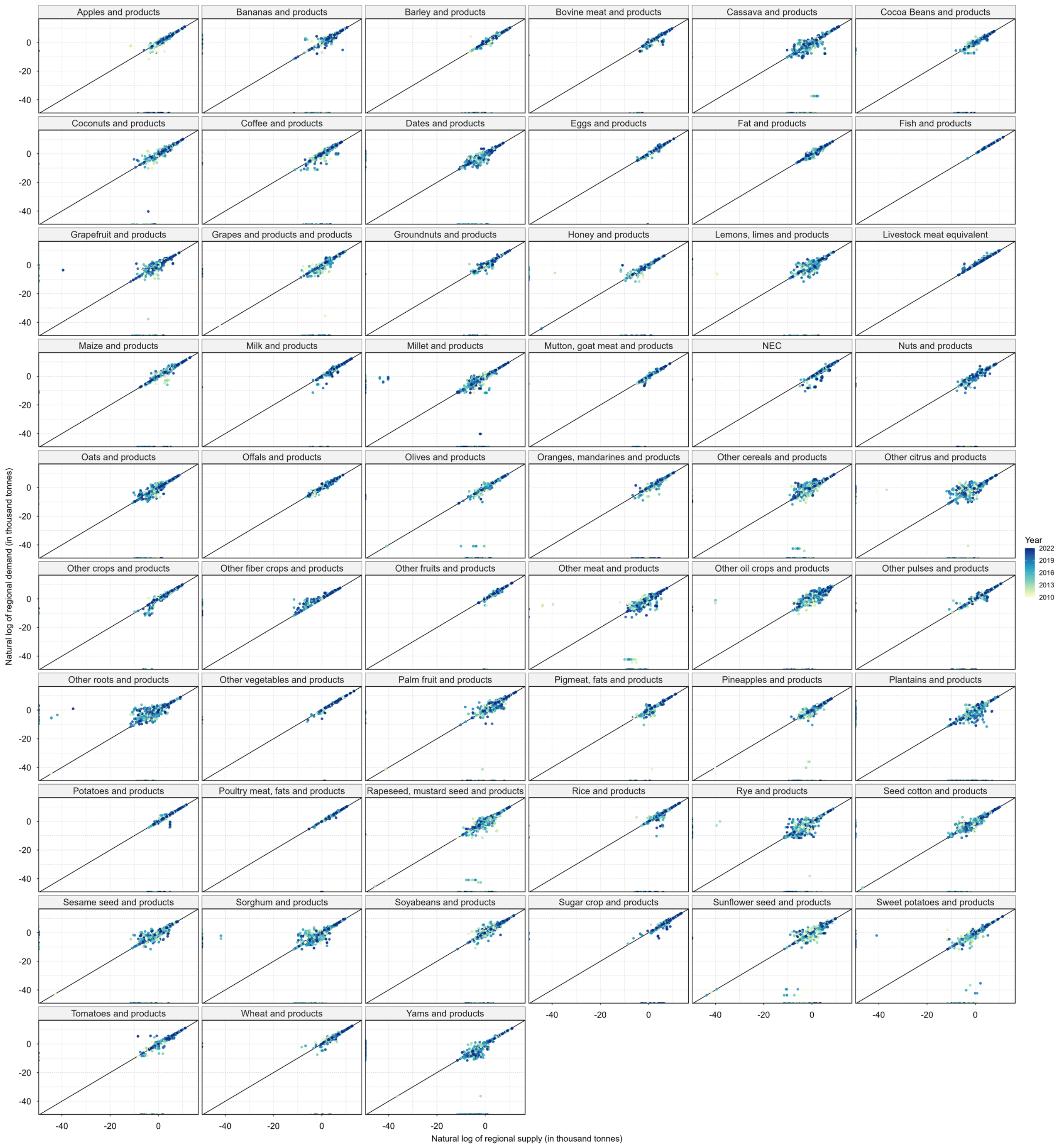

Supplementary Fig. S4 | Balance check for regional supply and demand in the T-FBS by aggregated PCe commodities. Each point compares the total regional demand (y-axis) and total regional supply (x-axis) (both in thousand tonnes and natural logarithmic values) for each country and year (colors), grouped by aggregated PCe commodity (panels). Natural logarithmic values are used for both axes. A 45-degree reference line indicates perfect agreement.

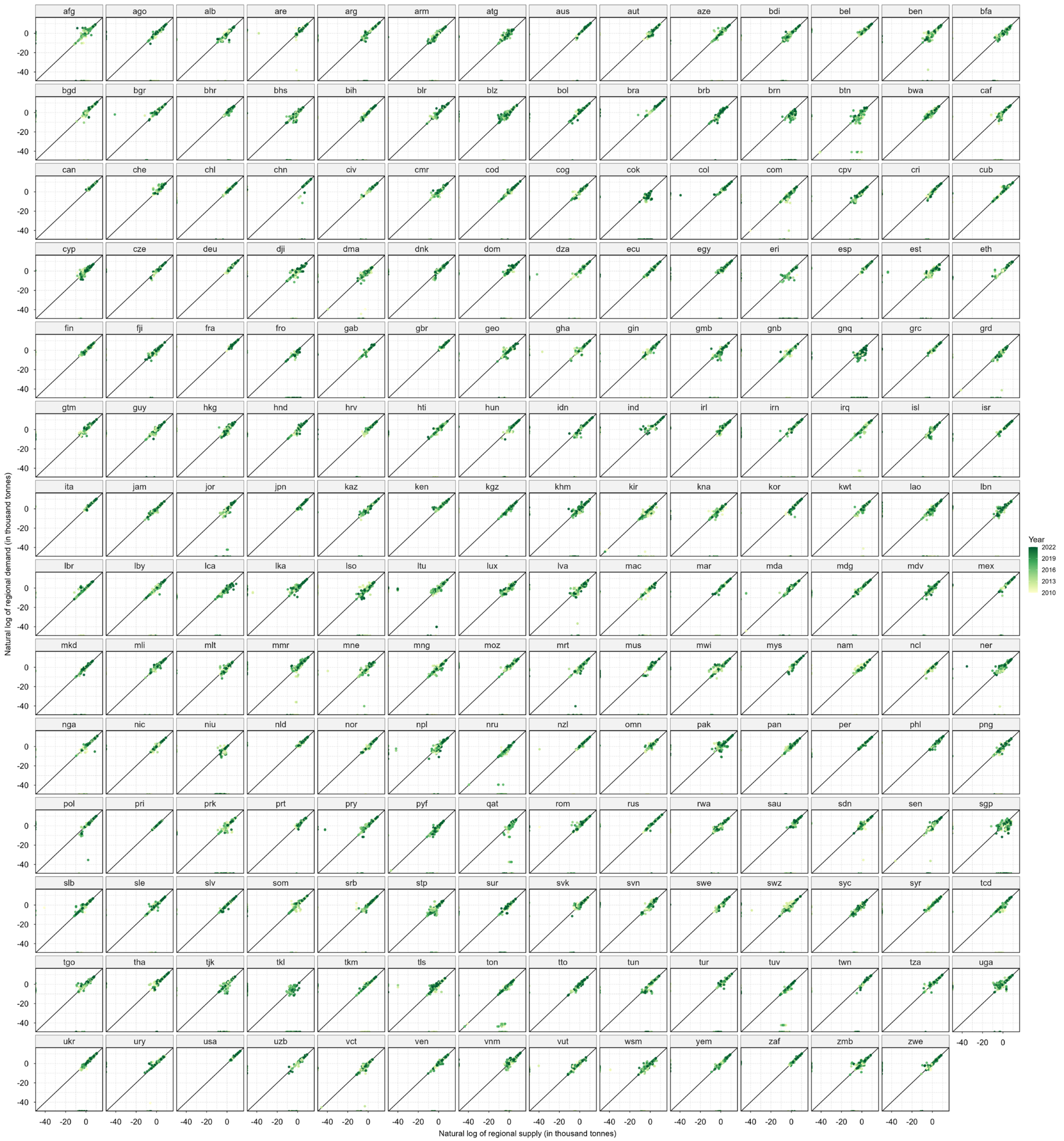

Supplementary Fig. S5 | Balance check for regional supply and demand in the T-FBS by countries (ISO). Each point compares the total regional demand (y-axis) and total regional supply (x-axis) (both in thousand tonnes and natural logarithmic values) for each aggregated PCe commodity and year (colors), grouped by countries/ISO (panels). Natural logarithmic values are used for both axes. A 45-degree reference line indicates perfect agreement.

Supplementary Table S1 | Mapping of items in the detailed Supply Utilization Account (SUA) dataset to aggregated Primary Commodity equivalent (PCe) commodities in the new traceable Food Balance Sheets (T-FBS). The source and sink SUA items follow consistent definitions provided in the FAO SUA or FBS datasets. However, several nonfood items may not be available in the FAO datasets, such as some oilseed cakes, fiber, and livestock meat equivalents, were generated in *gcamfaostat*. Items under "Livestock meat equivalent" were derived by converting live animal stocks to their meat equivalent using carcass yield. SUA items classified under NEC (Not Elsewhere Categorized) were aggregated for completeness. Note that this mapping is developed mainly based on definitions of FAO items[1]. The PCe aggregation is performed for each "PCe item" by aggregating sink SUA items into source SUA items per nest level. The nest levels represent the order of aggregation, starting with the largest nest level (indicating more processed commodities) and proceeding backward to the smallest nest level. When the nest level is 0, no aggregation is required, as the commodities are already classified as primary. Each "PCe item" (84, if not including NEC item) is further aggregated into an "Aggregated PCe item" (56, if not including NEC) to simplify the data in the T-FBS, while intermediate data from the processing stages remain accessible. This table is also archived in the data record "*Nested_Mapping_SUA_To_Traceable_FBS.csv*," which also includes FAO item codes. The mapping corresponds to the "*FAOSTAT/Mapping_gcamdata_SUA_PrimaryEquivalent.csv*" file used in *gcamfaostat*. Users may modify the mapping file to include more detailed data (e.g., setting nest levels to 0 and source item to be the same as sink item to keep SUA items from the PCe aggregation). Note that the term "item" (or "item code") used in FAOSTAT datasets is interchangeable with "commodity" in the context of this study. Consistent with FAO conventions, "and products" is appended to the item name to indicate that the commodity is a PCe commodity (they are included for "aggregated PCe" items but omitted for "PCe items" for simplicity).

Aggregated PCe item	PCe item	Nest level	Source SUA item	Sink SUA item
Wheat and products	Wheat	1	Wheat	Food preparations of flour, meal or malt extract
**Wheat and products**	Wheat	1	Wheat	Bulgur
**Wheat and products**	Wheat	1	Wheat	Breakfast cereals
**Wheat and products**	Wheat	1	Wheat	Bran of wheat
**Wheat and products**	Wheat	1	Wheat	Germ of wheat
**Wheat and products**	Wheat	1	Wheat	Wheat and meslin flour
**Wheat and products**	Wheat	2	Wheat and meslin flour	Wheat gluten
**Wheat and products**	Wheat	2	Wheat and meslin flour	Starch of wheat
**Wheat and products**	Wheat	2	Wheat and meslin flour	Bread
**Wheat and products**	Wheat	2	Wheat and meslin flour	Pastry
**Wheat and products**	Wheat	2	Wheat and meslin flour	Uncooked pasta, not stuffed or otherwise prepared
**Wheat and products**	Wheat	2	Wheat and meslin flour	Communion wafers, empty cachets of a kind suitable for pharmaceutical use, sealing wafers, rice paper and similar products.
**Wheat and products**	Wheat	2	Wheat and meslin flour	Mixes and doughs for the preparation of bakers wares
**Rice and products**	Rice	1	Rice	Rice, milled
**Rice and products**	Rice	1	Rice	Husked rice
**Rice and products**	Rice	1	Rice	Bran of rice
**Rice and products**	Rice	2	Husked rice	Rice, milled (husked)
**Rice and products**	Rice	3	Rice, milled	Rice, broken
**Rice and products**	Rice	4	Rice, broken	Flour of rice
**Rice and products**	Rice	4	Rice, broken	Starch of rice
**Rice and products**	Rice	4	Rice, broken	Rice, gluten
**Rice and products**	Rice	5	Bran of rice	Oil of rice bran
**Rice and products**	Rice	5	Bran of rice	Cake of rice bran
**Maize and products**	Corn	1	Maize (corn)	Flour of maize
**Maize and products**	Corn	1	Maize (corn)	Germ of maize
**Maize and products**	Corn	1	Maize (corn)	Bran of maize
**Maize and products**	Corn	2	Flour of maize	Starch of maize
**Maize and products**	Corn	2	Flour of maize	Maize gluten
**Maize and products**	Corn	3	Bran of maize	Gluten feed and meal
**Maize and products**	Corn	3	Maize gluten	Gluten feed and meal
**Maize and products**	Corn	4	Germ of maize	Oil of maize

[1] The "FBS and SUA list" from FAOSTAT can be downloaded from https://files-faostat.fao.org/production/FBS/FBS%20and%20SUA%20list.xlsx.

**Maize and products**	Corn	4	Germ of maize	Cake of maize
**Barley and products**	Barley	1	Barley	Pot barley
**Barley and products**	Barley	1	Barley	Bran of barley
**Barley and products**	Barley	1	Barley	Malt, whether or not roasted
**Barley and products**	Barley	2	Pot barley	Barley, pearled
**Barley and products**	Barley	2	Pot barley	Barley flour and grits
**Barley and products**	Barley	3	Malt, whether or not roasted	Beer of barley, malted
**Barley and products**	Barley	3	Malt, whether or not roasted	Malt extract
**Millet and products**	Millet	1	Millet	Flour of millet
**Millet and products**	Millet	1	Millet	Bran of millet
**Rye and products**	Rye	1	Rye	Flour of rye
**Rye and products**	Rye	1	Rye	Bran of rye
**Oats and products**	Oats	1	Oats	Oats, rolled
**Oats and products**	Oats	1	Oats	Bran of oats
**Sorghum and products**	Sorghum	1	Sorghum	Flour of sorghum
**Sorghum and products**	Sorghum	1	Sorghum	Bran of sorghum
**Other cereals and products**	CerealsNES	0	Quinoa	Quinoa
**Other cereals and products**	CerealsNES	0	Canary seed	Canary seed
**Other cereals and products**	CerealsNES	1	Buckwheat	Flour of buckwheat
**Other cereals and products**	CerealsNES	1	Buckwheat	Bran of buckwheat
**Other cereals and products**	CerealsNES	2	Fonio	Flour of fonio
**Other cereals and products**	CerealsNES	2	Fonio	Bran of fonio
**Other cereals and products**	CerealsNES	3	Triticale	Flour of triticale
**Other cereals and products**	CerealsNES	3	Triticale	Bran of triticale
**Other cereals and products**	CerealsNES	4	Mixed grain	Flour of mixed grain
**Other cereals and products**	CerealsNES	4	Mixed grain	Bran of mixed grain
**Other cereals and products**	CerealsNES	5	Cereals n.e.c.	Flour of cereals n.e.c.
**Other cereals and products**	CerealsNES	5	Cereals n.e.c.	Bran of cereals n.e.c.
**Other cereals and products**	CerealsNES	5	Cereals n.e.c.	Cereal preparations
**Soyabeans and products**	Soybean	1	Soya beans	Soya bean oil
**Soyabeans and products**	Soybean	1	Soya beans	Cake of soya beans
**Soyabeans and products**	Soybean	1	Soya beans	Soya sauce
**Soyabeans and products**	Soybean	1	Soya beans	Soya paste
**Soyabeans and products**	Soybean	1	Soya beans	Soya curd
**Palm fruit and products**	OilPalm	1	Oil palm fruit	Palm oil
**Palm fruit and products**	OilPalm	1	Oil palm fruit	Palm kernels
**Palm fruit and products**	OilPalm	2	Palm kernels	Oil of palm kernel
**Palm fruit and products**	OilPalm	2	Palm kernels	Cake of palm kernel
**Sunflower seed and products**	Sunflower seed	1	Sunflower seed	Sunflower-seed oil, crude
**Sunflower seed and products**	Sunflower seed	1	Sunflower seed	Cake of sunflower seed
**Rapeseed, mustard seed and products**	Rapeseed	1	Rape or colza seed	Rapeseed or canola oil, crude
**Rapeseed, mustard seed and products**	Rapeseed	1	Rape or colza seed	Cake of rapeseed
**Rapeseed, mustard seed and products**	Mustard seed	1	Mustard seed	Mustard seed oil, crude
**Rapeseed, mustard seed and products**	Mustard seed	1	Mustard seed	Flour of mustard seed
**Rapeseed, mustard seed and products**	Mustard seed	1	Mustard seed	Cake of mustard seed
**Coconuts and products**	Coconuts	1	Coconuts, in shell	Copra
**Coconuts and products**	Coconuts	1	Coconuts, in shell	Coconuts, desiccated
**Coconuts and products**	Coconuts	1	Coconuts, in shell	Coir, raw
**Coconuts and products**	Coconuts	2	Copra	Coconut oil
**Coconuts and products**	Coconuts	2	Copra	Cake of copra
**Sesame seed and products**	Sesame seed	1	Sesame seed	Oil of sesame seed
**Sesame seed and products**	Sesame seed	1	Sesame seed	Cake of sesame seed
**Olives and products**	Olives	1	Olives	Olives preserved
**Olives and products**	Olives	1	Olives	Olive oil
**Olives and products**	Olives	1	Olives	Oil of olive residues
**Olives and products**	Olives	1	Olives	Cake, olive residues
**Other oil crops and products**	Linseed	1	Linseed	Oil of linseed
**Other oil crops and products**	Linseed	1	Linseed	Cake of linseed
**Other oil crops and products**	Castor oil seed	1	Castor oil seeds	Oil of castor beans
**Other oil crops and products**	Castor oil seed	2	Oil of castor beans	Castor oil, hydrogenated
**Other oil crops and products**	Tallowtree seed	1	Tallowtree seeds	Vegetable tallow
**Other oil crops and products**	Karite nuts (sheanuts)	1	Karite nuts (sheanuts)	Butter of karite nuts
**Other oil crops and products**	Kapok fruit	1	Kapok fruit	Kapokseed in shell
**Other oil crops and products**	Kapok fruit	1	Kapok fruit	Kapok fibre, raw
**Other oil crops and products**	Kapok fruit	2	Kapokseed in shell	Kapokseed, shelled
**Other oil crops and products**	Kapok fruit	3	Kapokseed, shelled	Oil of kapok

**Other oil crops and products**	Kapok fruit	3	Kapokseed, shelled	Cake of kapok
**Other oil crops and products**	OilCropNES	0	Melonseed	Melonseed
**Other oil crops and products**	OilCropNES	1	Safflower seed	Safflower-seed oil, crude
**Other oil crops and products**	OilCropNES	1	Safflower seed	Cake of safflowerseed
**Other oil crops and products**	OilCropNES	2	Jojoba seeds	Jojoba oil
**Other oil crops and products**	OilCropNES	2	Jojoba seeds	Cake, jojoba
**Other oil crops and products**	OilCropNES	3	Poppy seed	Oil of poppy seed
**Other oil crops and products**	OilCropNES	3	Poppy seed	Cake, poppy seed
**Other oil crops and products**	OilCropNES	4	Hempseed	Oil of hempseed
**Other oil crops and products**	OilCropNES	4	Hempseed	Cake of hempseed
**Other oil crops and products**	OilCropNES	5	Other oil seeds, n.e.c.	Other oil of vegetable origin, crude n.e.c.
**Other oil crops and products**	OilCropNES	5	Other oil seeds, n.e.c.	Cake, vegetable origin nes
**Other oil crops and products**	OilCropNES	5	Other oil seeds, n.e.c.	Flours and meals of oil seeds or oleaginous fruits, except those of mustard
**Other oil crops and products**	OilCropNES	6	Tung nuts	Oil of tung nuts
**Seed cotton and products**	Seed cotton	1	Seed cotton, unginned	Cotton seed
**Seed cotton and products**	Seed cotton	1	Seed cotton, unginned	Cotton lint, ginned
**Seed cotton and products**	Seed cotton	2	Cotton seed	Cottonseed oil
**Seed cotton and products**	Seed cotton	2	Cotton seed	Cake of cottonseed
**Seed cotton and products**	Seed cotton	2	Cotton seed	Fiber, cottonseed
**Other fiber crops and products**	FiberCropNES	0	Agave fibres, raw, n.e.c.	Agave fibres, raw, n.e.c.
**Other fiber crops and products**	FiberCropNES	0	Kenaf, and other textile bast fibres, raw or retted	Kenaf, and other textile bast fibres, raw or retted
**Other fiber crops and products**	FiberCropNES	0	Other fibre crops, raw, n.e.c.	Other fibre crops, raw, n.e.c.
**Other fiber crops and products**	FiberCropNES	0	Flax, raw or retted	Flax, raw or retted
**Other fiber crops and products**	FiberCropNES	0	True hemp, raw or retted	True hemp, raw or retted
**Other fiber crops and products**	FiberCropNES	0	Jute, raw or retted	Jute, raw or retted
**Other fiber crops and products**	FiberCropNES	0	Abaca, manila hemp, raw	Abaca, manila hemp, raw
**Other fiber crops and products**	FiberCropNES	0	Ramie, raw or retted	Ramie, raw or retted
**Other fiber crops and products**	FiberCropNES	0	Sisal, raw	Sisal, raw
**Groundnuts and products**	Groundnuts, with shell	1	Groundnuts, excluding shelled	Groundnuts, shelled
**Groundnuts and products**	Groundnuts, with shell	2	Groundnuts, shelled	Cake of groundnuts
**Groundnuts and products**	Groundnuts, with shell	2	Groundnuts, shelled	Prepared groundnuts
**Groundnuts and products**	Groundnuts, with shell	2	Groundnuts, shelled	Peanut butter
**Groundnuts and products**	Groundnuts, with shell	2	Groundnuts, shelled	Groundnut oil
**Nuts and products**	NutsSeedsNES	0	Chestnuts, in shell	Chestnuts, in shell
**Nuts and products**	NutsSeedsNES	0	Areca nuts	Areca nuts
**Nuts and products**	NutsSeedsNES	0	Kola nuts	Kola nuts
**Nuts and products**	NutsSeedsNES	1	Brazil nuts, in shell	Brazil nuts, shelled
**Nuts and products**	NutsSeedsNES	2	Walnuts, in shell	Walnuts, shelled
**Nuts and products**	NutsSeedsNES	3	Cashew nuts, in shell	Cashew nuts, shelled
**Nuts and products**	NutsSeedsNES	4	Almonds, in shell	Almonds, shelled
**Nuts and products**	NutsSeedsNES	5	Hazelnuts, in shell	Hazelnuts, shelled
**Nuts and products**	NutsSeedsNES	6	Brazil nuts, shelled	Prepared nuts
**Nuts and products**	NutsSeedsNES	6	Walnuts, shelled	Prepared nuts
**Nuts and products**	NutsSeedsNES	6	Cashew nuts, shelled	Prepared nuts
**Nuts and products**	NutsSeedsNES	6	Other nuts (excluding wild edible nuts and groundnuts), in shell, n.e.c.	Prepared nuts
**Nuts and products**	NutsSeedsNES	6	Pistachios, in shell	Prepared nuts
**Nuts and products**	NutsSeedsNES	6	Almonds, shelled	Prepared nuts
**Nuts and products**	NutsSeedsNES	6	Hazelnuts, shelled	Prepared nuts
**Other pulses and products**	Legumes	1	Beans, dry	Flour of pulses
**Other pulses and products**	Legumes	1	Peas, dry	Flour of pulses
**Other pulses and products**	Legumes	1	Broad beans and horse beans, dry	Flour of pulses
**Other pulses and products**	Legumes	1	Chick peas, dry	Flour of pulses
**Other pulses and products**	Legumes	1	Lentils, dry	Flour of pulses
**Other pulses and products**	Legumes	1	Cow peas, dry	Flour of pulses

**Other pulses and products**	Legumes	1	Pigeon peas, dry	Bran of pulses
**Other pulses and products**	Legumes	1	Bambara beans, dry	Bran of pulses
**Other pulses and products**	Legumes	1	Vetches	Bran of pulses
**Other pulses and products**	Legumes	1	Lupins	Bran of pulses
**Other pulses and products**	Legumes	1	Other pulses n.e.c.	Bran of pulses
**Potatoes and products**	Potatoes	1	Potatoes	Potatoes, frozen
**Potatoes and products**	Potatoes	1	Potatoes	Flour, meal, powder, flakes, granules and pellets of potatoes
**Potatoes and products**	Potatoes	1	Potatoes	Starch of potatoes
**Potatoes and products**	Potatoes	1	Potatoes	Tapioca of potatoes
**Cassava and products**	Cassava	1	Cassava, fresh	Cassava, dry
**Cassava and products**	Cassava	1	Cassava, fresh	Flour of cassava
**Cassava and products**	Cassava	1	Cassava, fresh	Starch of cassava
**Cassava and products**	Cassava	1	Cassava, fresh	Tapioca of cassava
**Sweet potatoes and products**	Sweet potatoes	0	Sweet potatoes	Sweet potatoes
**Yams and products**	Yams	0	Yams	Yams
**Other roots and products**	Roots and tubers nes	0	Taro	Taro
**Other roots and products**	Roots and tubers nes	0	Yautia	Yautia
**Other roots and products**	Roots and tubers nes	1	Edible roots and tubers with high starch or inulin content, n.e.c., fresh	Edible roots and tubers with high starch or inulin content, n.e.c., dry
**Other roots and products**	Roots and tubers nes	1	Edible roots and tubers with high starch or inulin content, n.e.c., fresh	Flour of roots and tubers n.e.c.
**Sugar crop and products**	SugarCrop	1	Sugar cane	Cane sugar, non-centrifugal
**Sugar crop and products**	SugarCrop	1	Sugar cane	Raw cane or beet sugar (centrifugal only)
**Sugar crop and products**	SugarCrop	1	Sugar cane	Molasses
**Sugar crop and products**	SugarCrop	1	Sugar beet	Cane sugar, non-centrifugal
**Sugar crop and products**	SugarCrop	1	Sugar beet	Raw cane or beet sugar (centrifugal only)
**Sugar crop and products**	SugarCrop	1	Sugar beet	Molasses
**Sugar crop and products**	SugarCrop	1	Other sugar crops n.e.c.	Cane sugar, non-centrifugal
**Sugar crop and products**	SugarCrop	1	Other sugar crops n.e.c.	Raw cane or beet sugar (centrifugal only)
**Sugar crop and products**	SugarCrop	1	Other sugar crops n.e.c.	Molasses
**Sugar crop and products**	SugarCrop	2	Raw cane or beet sugar (centrifugal only)	Refined sugar
**Sugar crop and products**	SugarCrop	3	Refined sugar	Sugar confectionery
**Sugar crop and products**	SugarCrop	3	Refined sugar	Sugar and syrups n.e.c.
**Tomatoes and products**	Tomatoes	1	Tomatoes	Tomato juice
**Tomatoes and products**	Tomatoes	1	Tomatoes	Paste of tomatoes
**Tomatoes and products**	Tomatoes	1	Tomatoes	Tomatoes, peeled (o/t vinegar)
**Other vegetables and products**	Maize, green	1	Green corn (maize)	Sweet corn, frozen
**Other vegetables and products**	Maize, green	1	Green corn (maize)	Sweet corn, prepared or preserved
**Other vegetables and products**	Mushrooms and truffles	1	Mushrooms and truffles	Dried mushrooms
**Other vegetables and products**	Mushrooms and truffles	1	Mushrooms and truffles	Canned mushrooms
**Other vegetables and products**	VegetablesNES	0	Lettuce and chicory	Lettuce and chicory
**Other vegetables and products**	VegetablesNES	0	Cassava leaves	Cassava leaves
**Other vegetables and products**	VegetablesNES	1	Chillies and peppers, dry (Capsicum spp., Pimenta spp.), raw	Other vegetable juices
**Other vegetables and products**	VegetablesNES	1	Onions and shallots, dry (excluding dehydrated)	Vegetables, pulses and potatoes, preserved by vinegar or acetic acid
**Other vegetables and products**	VegetablesNES	1	Onions and shallots, green	Vegetables, dehydrated
**Other vegetables and products**	VegetablesNES	1	Asparagus	Other vegetables provisionally preserved
**Other vegetables and products**	VegetablesNES	1	Cabbages	Coffee substitutes
**Other vegetables and products**	VegetablesNES	1	Cauliflowers and broccoli	Homogenized vegetable preparations
**Other vegetables and products**	VegetablesNES	1	Spinach	Vegetables preserved nes (o/t vinegar)
**Other vegetables and products**	VegetablesNES	1	Artichokes	Vegetables frozen
**Other vegetables and products**	VegetablesNES	1	Watermelons	Vegetables preserved (frozen)

**Other vegetables and products**	VegetablesNES	1	Cantaloupes and other melons	Vegetables preserved (frozen)
**Other vegetables and products**	VegetablesNES	1	Chillies and peppers, green (Capsicum spp. and Pimenta spp.)	Vegetables preserved (frozen)
**Other vegetables and products**	VegetablesNES	1	Cucumbers and gherkins	Vegetables preserved (frozen)
**Other vegetables and products**	VegetablesNES	1	Eggplants (aubergines)	Vegetables preserved (frozen)
**Other vegetables and products**	VegetablesNES	1	Pumpkins, squash and gourds	Vegetables preserved (frozen)
**Other vegetables and products**	VegetablesNES	1	Peas, green	Vegetables preserved (frozen)
**Other vegetables and products**	VegetablesNES	1	Carrots and turnips	Vegetables preserved (frozen)
**Other vegetables and products**	VegetablesNES	1	Green garlic	Vegetables preserved (frozen)
**Other vegetables and products**	VegetablesNES	1	Leeks and other alliaceous vegetables	Vegetables preserved (frozen)
**Other vegetables and products**	VegetablesNES	1	Locust beans (carobs)	Vegetables preserved (frozen)
**Other vegetables and products**	VegetablesNES	1	Chicory roots	Vegetables preserved (frozen)
**Other vegetables and products**	VegetablesNES	1	Okra	Vegetables preserved (frozen)
**Other vegetables and products**	VegetablesNES	1	String beans	Vegetables preserved (frozen)
**Other vegetables and products**	VegetablesNES	1	Other beans, green	Vegetables preserved (frozen)
**Other vegetables and products**	VegetablesNES	1	Broad beans and horse beans, green	Vegetables preserved (frozen)
**Other vegetables and products**	VegetablesNES	1	Other vegetables, fresh n.e.c.	Vegetables preserved (frozen)
**Oranges, mandarines and products**	Oranges	1	Oranges	Orange juice
**Oranges, mandarines and products**	Oranges	1	Tangerines, mandarins, clementines	Orange juice, concentrated
**Oranges, mandarines and products**	Oranges	1	Tangerines, mandarins, clementines	Juice of tangerine
**Other citrus and products**	Fruit, citrus nes	1	Other citrus fruit, n.e.c.	Juice of citrus fruit n.e.c.
**Other citrus and products**	Fruit, citrus nes	1	Other citrus fruit, n.e.c.	Citrus juice, concentrated n.e.c.
**Lemons, limes and products**	Lemons and limes	1	Lemons and limes	Juice of lemon
**Lemons, limes and products**	Lemons and limes	1	Lemons and limes	Lemon juice, concentrated
**Grapefruit and products**	Grapefruit	1	Pomelos and grapefruits	Grapefruit juice
**Grapefruit and products**	Grapefruit	1	Pomelos and grapefruits	Grapefruit juice, concentrated
**Bananas and products**	Bananas	0	Bananas	Bananas
**Plantains and products**	Plantains and others	0	Plantains and cooking bananas	Plantains and cooking bananas
**Dates and products**	Dates	0	Dates	Dates
**Apples and products**	Apples	1	Apples	Apple juice
**Apples and products**	Apples	1	Apples	Apple juice, concentrated
**Pineapples and products**	Pineapples	1	Pineapples	Pineapple juice
**Pineapples and products**	Pineapples	1	Pineapples	Pineapples, otherwise prepared or preserved
**Pineapples and products**	Pineapples	1	Pineapples	Juice of pineapples, concentrated
**Grapes and products and products**	Grapes	1	Grapes	Raisins
**Grapes and products and products**	Grapes	1	Grapes	Grape juice
**Grapes and products and products**	Grapes	1	Grapes	Must of grape
**Other fruits and products**	FruitsNES	0	Pears	Pears
**Other fruits and products**	FruitsNES	0	Papayas	Papayas
**Other fruits and products**	FruitsNES	0	Peaches and nectarines	Peaches and nectarines
**Other fruits and products**	FruitsNES	0	Avocados	Avocados
**Other fruits and products**	FruitsNES	1	Sour cherries	Fruit, nuts, peel, sugar preserved
**Other fruits and products**	FruitsNES	1	Cherries	Homogenized cooked fruit, prepared
**Other fruits and products**	FruitsNES	1	Strawberries	Other tropical fruit, dried
**Other fruits and products**	FruitsNES	1	Kiwi fruit	Other fruit n.e.c., dried
**Other fruits and products**	FruitsNES	1	Quinces	Juice of fruits n.e.c.
**Other fruits and products**	FruitsNES	1	Currants	Flour of fruits
**Other fruits and products**	FruitsNES	1	Gooseberries	Fruit prepared n.e.c.
**Other fruits and products**	FruitsNES	1	Raspberries	Fruit prepared n.e.c.

**Other fruits and products**	FruitsNES	1	Blueberries	Fruit prepared n.e.c.
**Other fruits and products**	FruitsNES	1	Cranberries	Fruit prepared n.e.c.
**Other fruits and products**	FruitsNES	1	Other berries and fruits of the genus vaccinium n.e.c.	Fruit prepared n.e.c.
**Other fruits and products**	FruitsNES	1	Persimmons	Fruit prepared n.e.c.
**Other fruits and products**	FruitsNES	1	Cashewapple	Fruit prepared n.e.c.
**Other fruits and products**	FruitsNES	1	Other fruits, n.e.c.	Fruit prepared n.e.c.
**Other fruits and products**	FruitsNES	1	Other tropical fruits, n.e.c.	Fruit prepared n.e.c.
**Other fruits and products**	FruitsNES	1	Other pome fruits	Fruit prepared n.e.c.
**Other fruits and products**	FruitsNES	1	Other stone fruits	Fruit prepared n.e.c.
**Other fruits and products**	FruitsNES	2	Apricots	Apricots, dried
**Other fruits and products**	FruitsNES	2	Figs	Figs, dried
**Other fruits and products**	FruitsNES	3	Mangoes, guavas and mangosteens	Juice of mango
**Other fruits and products**	FruitsNES	4	Plums and sloes	Plums, dried
**Other fruits and products**	FruitsNES	4	Plums and sloes	Juice of plum
**Other fruits and products**	FruitsNES	4	Plums and sloes	Juice of plum, concentrated
**Coffee and products**	Coffee, green	1	Coffee, green	Coffee, decaffeinated or roasted
**Coffee and products**	Coffee, green	1	Coffee, green	Coffee extracts
**Cocoa Beans and products**	Cocoa, beans	1	Cocoa beans	Cocoa paste not defatted
**Cocoa Beans and products**	Cocoa, beans	2	Cocoa paste not defatted	Cocoa butter, fat and oil
**Cocoa Beans and products**	Cocoa, beans	2	Cocoa paste not defatted	Cocoa powder and cake
**Cocoa Beans and products**	Cocoa, beans	3	Cocoa butter, fat and oil	Chocolate products nes
**Cocoa Beans and products**	Cocoa, beans	3	Cocoa powder and cake	Chocolate products nes
**Other crops and products**	MiscCropNES	0	Tea leaves	Tea leaves
**Other crops and products**	MiscCropNES	0	Mate leaves	Mate leaves
**Other crops and products**	MiscCropNES	0	Pepper (Piper spp.), raw	Pepper (Piper spp.), raw
**Other crops and products**	MiscCropNES	0	Cloves (whole stems), raw	Cloves (whole stems), raw
**Other crops and products**	MiscCropNES	0	Nutmeg, mace, cardamoms, raw	Nutmeg, mace, cardamoms, raw
**Other crops and products**	MiscCropNES	0	Anise, badian, coriander, cumin, caraway, fennel and juniper berries, raw	Anise, badian, coriander, cumin, caraway, fennel and juniper berries, raw
**Other crops and products**	MiscCropNES	0	Cinnamon and cinnamon-tree flowers, raw	Cinnamon and cinnamon-tree flowers, raw
**Other crops and products**	MiscCropNES	0	Ginger, raw	Ginger, raw
**Other crops and products**	MiscCropNES	0	Vanilla, raw	Vanilla, raw
**Other crops and products**	MiscCropNES	0	Other stimulant, spice and aromatic crops, n.e.c.	Other stimulant, spice and aromatic crops, n.e.c.
**Other crops and products**	MiscCropNES	0	Peppermint, spearmint	Peppermint, spearmint
**Other crops and products**	MiscCropNES	0	Hop cones	Hop cones
**Other crops and products**	MiscCropNES	0	Unmanufactured tobacco	Unmanufactured tobacco
**Other crops and products**	MiscCropNES	0	Natural rubber in primary forms	Natural rubber in primary forms
**Other crops and products**	MiscCropNES	0	Pyrethrum, dried flowers	Pyrethrum, dried flowers
**Livestock meat equivalent**	BeefAnMeatEq	0	AnMeatEq, cattle	AnMeatEq, cattle
**Livestock meat equivalent**	BeefAnMeatEq	0	AnMeatEq, buffalo	AnMeatEq, buffalo
**Bovine meat and products**	Beef	1	Meat of cattle with the bone, fresh or chilled	Bovine meat, salted, dried or smoked
**Bovine meat and products**	Beef	1	Meat of cattle with the bone, fresh or chilled	Extracts and juices of meat, fish, crustaceans, molluscs or other aquatic invertebrates
**Bovine meat and products**	Beef	1	Meat of cattle with the bone, fresh or chilled	Meat of cattle boneless, fresh or chilled
**Bovine meat and products**	Beef	1	Meat of cattle with the bone, fresh or chilled	Sausages and similar products of meat, offal or blood of beef and veal
**Bovine meat and products**	Beef	1	Meat of cattle with the bone, fresh or chilled	Homogenized meat preparations
**Bovine meat and products**	Beef	1	Meat of cattle with the bone, fresh or chilled	beef and veal preparations nes
**Bovine meat and products**	Beef	1	Meat of cattle with the bone, fresh or chilled	Cattle, butcher fat
**Bovine meat and products**	Beef	1	Meat of buffalo, fresh or chilled	Bovine meat, salted, dried or smoked
**Bovine meat and products**	Beef	1	Meat of buffalo, fresh or chilled	Extracts and juices of meat, fish, crustaceans, molluscs or other aquatic invertebrates
**Bovine meat and products**	Beef	1	Meat of buffalo, fresh or chilled	Meat of cattle boneless, fresh or chilled
**Bovine meat and products**	Beef	1	Meat of buffalo, fresh or chilled	Sausages and similar products of meat, offal or blood of beef and veal
**Bovine meat and products**	Beef	1	Meat of buffalo, fresh or chilled	Homogenized meat preparations
**Bovine meat and products**	Beef	1	Meat of buffalo, fresh or chilled	beef and veal preparations nes
**Bovine meat and products**	Beef	1	Meat of buffalo, fresh or chilled	Cattle, butcher fat
**Bovine meat and products**	Beef	2	Cattle, butcher fat	Tallow

**Milk and products**	Milk, whole fresh cow	1	Raw milk of cattle	Skim milk of cows
**Milk and products**	Milk, whole fresh cow	1	Raw milk of cattle	Whole milk powder
**Milk and products**	Milk, whole fresh cow	1	Raw milk of cattle	Whole milk, evaporated
**Milk and products**	Milk, whole fresh cow	1	Raw milk of cattle	Whole milk, condensed
**Milk and products**	Milk, whole fresh cow	1	Raw milk of cattle	Yoghurt
**Milk and products**	Milk, whole fresh cow	1	Raw milk of cattle	Whey, fresh
**Milk and products**	Milk, whole fresh cow	1	Raw milk of cattle	Cheese from whole cow milk
**Milk and products**	Milk, whole fresh cow	1	Raw milk of cattle	Cream, fresh
**Milk and products**	Milk, whole fresh cow	1	Raw milk of cattle	Butter of cow milk
**Milk and products**	Milk, whole fresh cow	1	Raw milk of cattle	Ghee from cow milk
**Milk and products**	Milk, whole fresh cow	2	Cream, fresh	Ice cream and other edible ice
**Milk and products**	Milk, whole fresh cow	2	Cream, fresh	Processed cheese
**Milk and products**	Milk, whole fresh cow	3	Skim milk of cows	Cheese from skimmed cow milk
**Milk and products**	Milk, whole fresh cow	3	Skim milk of cows	Skim milk, condensed
**Milk and products**	Milk, whole fresh cow	3	Skim milk of cows	Skim milk, evaporated
**Milk and products**	Milk, whole fresh cow	3	Skim milk of cows	Skim milk and whey powder
**Milk and products**	Milk, whole fresh cow	3	Skim milk of cows	Buttermilk, dry
**Milk and products**	Milk, whole fresh cow	3	Skim milk of cows	Casein
**Milk and products**	Milk, whole fresh camel	0	Raw milk of camel	Raw milk of camel
**Milk and products**	Milk, whole fresh buffalo	1	Raw milk of buffalo	Ghee from buffalo milk
**Milk and products**	Milk, whole fresh buffalo	1	Raw milk of buffalo	Butter of buffalo milk
**Milk and products**	Milk, whole fresh buffalo	1	Raw milk of buffalo	Cheese from milk of buffalo, fresh or processed
**Milk and products**	Milk, whole fresh buffalo	1	Raw milk of buffalo	Skim milk of buffalo
**Milk and products**	Milk, whole fresh goat	1	Raw milk of goats	Butter of goat milk
**Milk and products**	Milk, whole fresh goat	1	Raw milk of goats	Cheese from milk of goats, fresh or processed
**Milk and products**	Milk, whole fresh goat	1	Raw milk of goats	Skim milk of goat
**Milk and products**	Milk, whole fresh sheep	1	Raw milk of sheep	Butter and ghee of sheep milk
**Milk and products**	Milk, whole fresh sheep	1	Raw milk of sheep	Cheese from milk of sheep, fresh or processed
**Milk and products**	Milk, whole fresh sheep	1	Raw milk of sheep	Skim sheep milk
**Livestock meat equivalent**	PorkAnMeatEq	0	AnMeatEq, pig	AnMeatEq, pig
**Pigmeat, fats and products**	Pork	1	Meat of pig with the bone, fresh or chilled	Meat of pig boneless, fresh or chilled
**Pigmeat, fats and products**	Pork	1	Meat of pig with the bone, fresh or chilled	Sausages and similar products of meat, offal or blood of pig
**Pigmeat, fats and products**	Pork	1	Meat of pig with the bone, fresh or chilled	Pig meat preparations
**Pigmeat, fats and products**	Pork	1	Meat of pig with the bone, fresh or chilled	Pig meat, cuts, salted, dried or smoked (bacon and ham)
**Pigmeat, fats and products**	Pork	1	Meat of pig with the bone, fresh or chilled	Pig, butcher fat
**Pigmeat, fats and products**	Pork	2	Pig, butcher fat	Lard stearine and lard oil
**Pigmeat, fats and products**	Pork	2	Pig, butcher fat	Pig fat, rendered
**Livestock meat equivalent**	PoultryAnMeatEq	0	AnMeatEq, duck	AnMeatEq, duck
**Livestock meat equivalent**	PoultryAnMeatEq	0	AnMeatEq, chicken	AnMeatEq, chicken
**Livestock meat equivalent**	PoultryAnMeatEq	0	AnMeatEq, goose and guinea fowl	AnMeatEq, goose and guinea fowl
**Livestock meat equivalent**	PoultryAnMeatEq	0	AnMeatEq, turkey	AnMeatEq, turkey
**Poultry meat, fats and products**	PoultryMeat	0	Meat of ducks, fresh or chilled	Meat of ducks, fresh or chilled
**Poultry meat, fats and products**	PoultryMeat	0	Meat of geese, fresh or chilled	Meat of geese, fresh or chilled
**Poultry meat, fats and products**	PoultryMeat	0	Meat of turkeys, fresh or chilled	Meat of turkeys, fresh or chilled
**Poultry meat, fats and products**	PoultryMeat	1	Meat of chickens, fresh or chilled	Fatty liver preparations
**Poultry meat, fats and products**	PoultryMeat	1	Meat of chickens, fresh or chilled	Poultry meat preparations
**Poultry meat, fats and products**	PoultryMeat	1	Meat of chickens, fresh or chilled	Fat of poultry
**Poultry meat, fats and products**	PoultryMeat	2	Fat of poultry	Poultry fat, rendered
**Eggs and products**	PoultryEgg	0	Eggs from other birds in shell, fresh, n.e.c.	Eggs from other birds in shell, fresh, n.e.c.

**Eggs and products**	PoultryEgg	1	Hen eggs in shell, fresh	Egg albumin
**Eggs and products**	PoultryEgg	1	Hen eggs in shell, fresh	Eggs, liquid
**Eggs and products**	PoultryEgg	1	Hen eggs in shell, fresh	Eggs, dried
**Livestock meat equivalent**	SheepGoatAnMeatEq	0	AnMeatEq, sheep	AnMeatEq, sheep
**Livestock meat equivalent**	SheepGoatAnMeatEq	0	AnMeatEq, goat	AnMeatEq, goat
**Mutton, goat meat and products**	SheepGoat	0	Meat of sheep, fresh or chilled	Meat of sheep, fresh or chilled
**Mutton, goat meat and products**	SheepGoat	0	Meat of goat, fresh or chilled	Meat of goat, fresh or chilled
**Fish and products**	Freshwater Fish	0	Freshwater Fish	Freshwater Fish
**Fish and products**	Fish, Body Oil	0	Fish, Body Oil	Fish, Body Oil
**Fish and products**	Fish, Liver Oil	0	Fish, Liver Oil	Fish, Liver Oil
**Fish and products**	Demersal Fish	0	Demersal Fish	Demersal Fish
**Fish and products**	Pelagic Fish	0	Pelagic Fish	Pelagic Fish
**Fish and products**	Marine Fish, Other	0	Marine Fish, Other	Marine Fish, Other
**Fish and products**	Crustaceans	0	Crustaceans	Crustaceans
**Fish and products**	Cephalopods	0	Cephalopods	Cephalopods
**Fish and products**	Molluscs, Other	0	Molluscs, Other	Molluscs, Other
**Fish and products**	Aquatic Animals, Others	0	Aquatic Animals, Others	Aquatic Animals, Others
**Fish and products**	Aquatic Plants	0	Aquatic Plants	Aquatic Plants
**Fish and products**	Meat, Aquatic Mammals	0	Meat, Aquatic Mammals	Meat, Aquatic Mammals
**Livestock meat equivalent**	OtherMeatAnMeatEq	0	AnMeatEq, rabbit	AnMeatEq, rabbit
**Livestock meat equivalent**	OtherMeatAnMeatEq	0	AnMeatEq, camel	AnMeatEq, camel
**Livestock meat equivalent**	OtherMeatAnMeatEq	0	AnMeatEq, other camelids	AnMeatEq, other camelids
**Livestock meat equivalent**	OtherMeatAnMeatEq	0	AnMeatEq, horse	AnMeatEq, horse
**Livestock meat equivalent**	OtherMeatAnMeatEq	0	AnMeatEq, ass	AnMeatEq, ass
**Livestock meat equivalent**	OtherMeatAnMeatEq	0	AnMeatEq, mule	AnMeatEq, mule
**Livestock meat equivalent**	OtherMeatAnMeatEq	0	AnMeatEq, other rodents	AnMeatEq, other rodents
**Other meat and products**	OtherMeatOther	0	Snails, fresh, chilled, frozen, dried, salted or in brine, except sea snails	Snails, fresh, chilled, frozen, dried, salted or in brine, except sea snails
**Other meat and products**	OtherMeatMeat	1	Meat of rabbits and hares, fresh or chilled	Meat prepared n.e.c.
**Other meat and products**	OtherMeatMeat	1	Meat of camels, fresh or chilled	Meat prepared n.e.c.
**Other meat and products**	OtherMeatMeat	1	Meat of other domestic camelids, fresh or chilled	Meat prepared n.e.c.
**Other meat and products**	OtherMeatMeat	1	Horse meat, fresh or chilled	Meat prepared n.e.c.
**Other meat and products**	OtherMeatMeat	1	Meat of asses, fresh or chilled	Meat prepared n.e.c.
**Other meat and products**	OtherMeatMeat	1	Meat of mules, fresh or chilled	Meat prepared n.e.c.
**Other meat and products**	OtherMeatMeat	1	Meat of other domestic rodents, fresh or chilled	Meat prepared n.e.c.
**Other meat and products**	OtherMeatMeat	1	Meat of pigeons and other birds n.e.c., fresh, chilled or frozen	Meat prepared n.e.c.
**Other meat and products**	OtherMeatMeat	1	Game meat, fresh, chilled or frozen	Meat prepared n.e.c.
**Other meat and products**	OtherMeatMeat	1	Other meat n.e.c. (excluding mammals), fresh, chilled or frozen	Meat prepared n.e.c.
**Other meat and products**	OtherMeatMeat	1	Meat of rabbits and hares, fresh or chilled	Other meat and edible meat offal, salted, in brine, dried or smoked; edible flours and meals of meat or meat offal
**Other meat and products**	OtherMeatMeat	1	Meat of camels, fresh or chilled	Other meat and edible meat offal, salted, in brine, dried or smoked; edible flours and meals of meat or meat offal
**Other meat and products**	OtherMeatMeat	1	Meat of other domestic camelids, fresh or chilled	Other meat and edible meat offal, salted, in brine, dried or smoked; edible flours and meals of meat or meat offal
**Other meat and products**	OtherMeatMeat	1	Horse meat, fresh or chilled	Other meat and edible meat offal, salted, in brine, dried or smoked; edible flours and meals of meat or meat offal
**Other meat and products**	OtherMeatMeat	1	Meat of asses, fresh or chilled	Other meat and edible meat offal, salted, in brine, dried or smoked; edible flours and meals of meat or meat offal
**Other meat and products**	OtherMeatMeat	1	Meat of mules, fresh or chilled	Other meat and edible meat offal, salted, in brine, dried or smoked; edible flours and meals of meat or meat offal
**Other meat and products**	OtherMeatMeat	1	Meat of other domestic rodents, fresh or chilled	Other meat and edible meat offal, salted, in brine, dried or smoked; edible flours and meals of meat or meat offal

**Other meat and products**	OtherMeatMeat	1	Meat of pigeons and other birds n.e.c., fresh, chilled or frozen	Other meat and edible meat offal, salted, in brine, dried or smoked; edible flours and meals of meat or meat offal
**Other meat and products**	OtherMeatMeat	1	Game meat, fresh, chilled or frozen	Other meat and edible meat offal, salted, in brine, dried or smoked; edible flours and meals of meat or meat offal
**Other meat and products**	OtherMeatMeat	1	Other meat n.e.c. (excluding mammals), fresh, chilled or frozen	Other meat and edible meat offal, salted, in brine, dried or smoked; edible flours and meals of meat or meat offal
**Offals and products**	OtherMeatOffals	0	Edible offal of cattle, fresh, chilled or frozen	Edible offal of cattle, fresh, chilled or frozen
**Offals and products**	OtherMeatOffals	0	Edible offal of buffalo, fresh, chilled or frozen	Edible offal of buffalo, fresh, chilled or frozen
**Offals and products**	OtherMeatOffals	0	Edible offal of pigs, fresh, chilled or frozen	Edible offal of pigs, fresh, chilled or frozen
**Offals and products**	OtherMeatOffals	0	Edible offal of sheep, fresh, chilled or frozen	Edible offal of sheep, fresh, chilled or frozen
**Offals and products**	OtherMeatOffals	0	Edible offal of goat, fresh, chilled or frozen	Edible offal of goat, fresh, chilled or frozen
**Offals and products**	OtherMeatOffals	0	Edible offals of horses and other equines, fresh, chilled or frozen	Edible offals of horses and other equines, fresh, chilled or frozen
**Offals and products**	OtherMeatOffals	0	Edible offals of camels and other camelids, fresh, chilled or frozen	Edible offals of camels and other camelids, fresh, chilled or frozen
**Offals and products**	OtherMeatOffals	0	Edible offals and liver of chickens and guinea fowl, fresh, chilled or frozen	Edible offals and liver of chickens and guinea fowl, fresh, chilled or frozen
**Offals and products**	OtherMeatOffals	0	Edible offals and liver of geese, fresh, chilled or frozen	Edible offals and liver of geese, fresh, chilled or frozen
**Offals and products**	OtherMeatOffals	0	Edible offals and liver of ducks, fresh, chilled or frozen	Edible offals and liver of ducks, fresh, chilled or frozen
**Offals and products**	OtherMeatOffals	0	Edible offals and liver of turkey, fresh, chilled or frozen	Edible offals and liver of turkey, fresh, chilled or frozen
**Offals and products**	OtherMeatOffals	0	Offals n.e.c. (excluding mammals), fresh, chilled or frozen	Offals n.e.c. (excluding mammals), fresh, chilled or frozen
**Offals and products**	OtherMeatOffals	0	Liver preparations	Liver preparations
**Fat and products**	OtherMeatFat	0	Cattle fat, unrendered	Cattle fat, unrendered
**Fat and products**	OtherMeatFat	0	Buffalo fat, unrendered	Buffalo fat, unrendered
**Fat and products**	OtherMeatFat	0	Sheep fat, unrendered	Sheep fat, unrendered
**Fat and products**	OtherMeatFat	0	Goat fat, unrendered	Goat fat, unrendered
**Fat and products**	OtherMeatFat	0	Fat of pigs	Fat of pigs
**Fat and products**	OtherMeatFat	0	Fat of camels	Fat of camels
**Fat and products**	OtherMeatFat	0	Fat of other camelids	Fat of other camelids
**Fat and products**	OtherMeatFat	0	Animal oils and fats n.e.c.	Animal oils and fats n.e.c.
**Fat and products**	OtherMeatFat	0	Degras	Degras
**Fat and products**	OtherMeatFat	0	Wool grease and lanolin	Wool grease and lanolin
**Fat and products**	OtherMeatFat	0	Fat preparations n.e.c.	Fat preparations n.e.c.
**Honey and products**	Honey and products	0	Natural honey	Natural honey
**NEC**	Beverages, fermented wheat	0	Wheat-fermented beverages	Wheat-fermented beverages
**NEC**	Beverages, fermented rice	0	Rice-fermented beverages	Rice-fermented beverages
**NEC**	Beer of maize	0	Beer of maize, malted	Beer of maize, malted
**NEC**	Beer of millet	0	Beer of millet, malted	Beer of millet, malted
**NEC**	Beer of sorghum	0	Beer of sorghum, malted	Beer of sorghum, malted
**NEC**	Infant food	0	Infant food	Infant food
**NEC**	Fructose chemically pure	0	Fructose, chemically pure	Fructose, chemically pure
**NEC**	Maltose chemically pure	0	Maltose, chemically pure	Maltose, chemically pure
**NEC**	Maple sugar and syrups	0	Refined cane or beet sugar, in solid form, containing added flavouring or colouring matter; maple sugar and maple syrup	Refined cane or beet sugar, in solid form, containing added flavouring or colouring matter; maple sugar and maple syrup
**NEC**	Fructose and syrup, other	0	Other fructose and syrup	Other fructose and syrup
**NEC**	Glucose and dextrose	0	Glucose and dextrose	Glucose and dextrose
**NEC**	Lactose	0	Lactose	Lactose
**NEC**	Isoglucose	0	Isoglucose	Isoglucose
**NEC**	Cake, others	0	Cake, oilseeds nes	Cake, oilseeds nes

**NEC**	Cider etc	0	Cider and other fermented beverages	Cider and other fermented beverages
**NEC**	Wine	0	Wine	Wine
**NEC**	Vermouths & similar	0	Vermouth and other wine of fresh grapes flavoured with plats or aromatic substances	Vermouth and other wine of fresh grapes flavoured with plats or aromatic substances
**NEC**	Alcohol non food	0	Undenatured ethyl alcohol of an alcoholic strength by volume of 80% vol or higher	Undenatured ethyl alcohol of an alcoholic strength by volume of 80% vol or higher
**NEC**	Beverages, distilled alcoholic	0	Undenatured ethyl alcohol of an alcoholic strength by volume of less than 80% vol; spirits, liqueurs and other spirituous beverages	Undenatured ethyl alcohol of an alcoholic strength by volume of less than 80% vol; spirits, liqueurs and other spirituous beverages
**NEC**	Tea, mate extracts	0	Extracts, essences and concentrates of tea or mate, and preparations with a basis thereof or with a basis of tea or mate	Extracts, essences and concentrates of tea or mate, and preparations with a basis thereof or with a basis of tea or mate
**NEC**	Gums, natural	0	Balata, gutta-percha, guayule, chicle and similar natural gums in primary forms or in plates, sheets or strip	Balata, gutta-percha, guayule, chicle and similar natural gums in primary forms or in plates, sheets or strip
**NEC**	Whey, condensed	0	Whey, condensed	Whey, condensed
**NEC**	Yoghurt, concentrated or not	0	Yoghurt, with additives	Yoghurt, with additives
**NEC**	Buttermilk, curdled, acidified milk	0	Buttermilk, curdled and acidified milk	Buttermilk, curdled and acidified milk
**NEC**	Whey, dry	0	Whey, dry	Whey, dry
**NEC**	Whey, cheese	0	Whey cheese	Whey cheese
**NEC**	Milk, products of natural constituents nes	0	Dairy products n.e.c.	Dairy products n.e.c.
**NEC**	Hides, cattle, fresh	0	Raw hides and skins of cattle	Raw hides and skins of cattle
**NEC**	Hides, buffalo, fresh	0	Raw hides and skins of buffaloes	Raw hides and skins of buffaloes
**NEC**	Wool, greasy	0	Shorn wool, greasy, including fleece-washed shorn wool	Shorn wool, greasy, including fleece-washed shorn wool
**NEC**	Skins, sheep, fresh	0	Raw hides and skins of sheep or lambs	Raw hides and skins of sheep or lambs
**NEC**	Skins, goat, fresh	0	Raw hides and skins of goats or kids	Raw hides and skins of goats or kids
**NEC**	Beeswax	0	Beeswax	Beeswax
**NEC**	Silk-worm cocoons, reelable	0	Silk-worm cocoons suitable for reeling	Silk-worm cocoons suitable for reeling
**NEC**	Silk, raw	0	Raw silk (not thrown)	Raw silk (not thrown)
**NEC**	Food prep nes	0	Food preparations n.e.c.	Food preparations n.e.c.
**NEC**	Margarine, liquid	0	Liquid margarine	Liquid margarine
**NEC**	Margarine, short	0	Margarine and shortening	Margarine and shortening
**NEC**	Oil, boiled etc	0	Animal or vegetable fats and oils and their fractions, chemically modified, except those hydrogenated, inter-esterified, re-esterified or elaidinized; inedible mixtures or preparations of animal or vegetable fats or oils	Animal or vegetable fats and oils and their fractions, chemically modified, except those hydrogenated, inter-esterified, re-esterified or elaidinized; inedible mixtures or preparations of animal or vegetable fats or oils
**NEC**	Oil, hydrogenated	0	hydrogenated oils and fats	hydrogenated oils and fats
**NEC**	Fatty acids	0	Industrial monocarboxylic fatty acids; acid oils from refining	Industrial monocarboxylic fatty acids; acid oils from refining
**NEC**	Fatty substance residues	0	Residues of fatty substances	Residues of fatty substances
**NEC**	Vegetable products, fresh or dry n.e.c.	0	Vegetable products, fresh or dry n.e.c.	Vegetable products, fresh or dry n.e.c.
**NEC**	Mango pulp	0	Mango pulp	Mango pulp
**NEC**	Other non-alcoholic caloric beverages	0	Other non-alcoholic caloric beverages	Other non-alcoholic caloric beverages
**NEC**	Cocoa husks and shells	0	Cocoa husks and shells	Cocoa husks and shells
**NEC**	Green tea (not fermented), black tea (fermented) and	0	Green tea (not fermented), black tea (fermented) and partly fermented tea, in	Green tea (not fermented), black tea (fermented) and partly fermented tea, in

	partly fermented tea, in immediate packings of a content not exceeding 3 kg		immediate packings of a content not exceeding 3 kg	immediate packings of a content not exceeding 3 kg
**NEC**	Standardized milk	0	Standardized milk	Standardized milk

Supplementary Table S2 | Regions in the new traceable Food Balance Sheets (FBS). The T-FBS dataset includes 195 regions, identified by ISO codes. This table provides the corresponding FAO area codes and names. For data processing convenience, Sudan (ISO: ssd; FAO area code: 276) and South Sudan (ssd; 277) are aggregated back into Sudan (Former) (sdn; 206). Additionally, 9 regions present in T-FBS (ISO: brn, cok, gnq, fro, niu, pri, eri, sgp, and tkl) were not included in FAO-FBS. This mapping corresponds to information in the "*FAOSTAT/Mapping_gcamdata_FAO_iso_reg.csv*" file used in *gcamfaostat*.

ISO	FAO Area Code	Name
arm	1	Armenia
afg	2	Afghanistan
alb	3	Albania
dza	4	Algeria
ago	7	Angola
atg	8	Antigua and Barbuda
arg	9	Argentina
aus	10	Australia
aut	11	Austria
bhs	12	Bahamas
bhr	13	Bahrain
brb	14	Barbados
bgd	16	Bangladesh
btn	18	Bhutan
bol	19	Bolivia (Plurinational State of)
bwa	20	Botswana
bra	21	Brazil
blz	23	Belize
slb	25	Solomon Islands
brn	26	Brunei Darussalam
bgr	27	Bulgaria
mmr	28	Myanmar
bdi	29	Burundi
cmr	32	Cameroon
can	33	Canada
cpv	35	Cabo Verde
caf	37	Central African Republic
lka	38	Sri Lanka
tcd	39	Chad
chl	40	Chile
chn	41	China, mainland
col	44	Colombia
com	45	Comoros
cog	46	Congo
cok	47	Cook Islands
cri	48	Costa Rica
cub	49	Cuba
cyp	50	Cyprus
aze	52	Azerbaijan
ben	53	Benin
dnk	54	Denmark
dma	55	Dominica
dom	56	Dominican Republic
blr	57	Belarus
ecu	58	Ecuador
egy	59	Egypt
slv	60	El Salvador
gnq	61	Equatorial Guinea
est	63	Estonia
fro	64	Faroe Islands
fji	66	Fiji
fin	67	Finland
fra	68	France
pyf	70	French Polynesia
dji	72	Djibouti
geo	73	Georgia
gab	74	Gabon
gmb	75	Gambia
deu	79	Germany
bih	80	Bosnia and Herzegovina

gha	81	Ghana
kir	83	Kiribati
grc	84	Greece
grd	86	Grenada
gtm	89	Guatemala
gin	90	Guinea
guy	91	Guyana
hti	93	Haiti
hnd	95	Honduras
hkg	96	China, Hong Kong SAR
hun	97	Hungary
hrv	98	Croatia
isl	99	Iceland
ind	100	India
idn	101	Indonesia
irn	102	Iran (Islamic Republic of)
irq	103	Iraq
irl	104	Ireland
isr	105	Israel
ita	106	Italy
civ	107	Cote dIvoire
kaz	108	Kazakhstan
jam	109	Jamaica
jpn	110	Japan
jor	112	Jordan
kgz	113	Kyrgyzstan
ken	114	Kenya
khm	115	Cambodia
prk	116	Democratic Peoples Republic of Korea
kor	117	Republic of Korea
kwt	118	Kuwait
lva	119	Latvia
lao	120	Lao Peoples Democratic Republic
lbn	121	Lebanon
lso	122	Lesotho
lbr	123	Liberia
lby	124	Libya
ltu	126	Lithuania
mac	128	China, Macao SAR
mdg	129	Madagascar
mwi	130	Malawi
mys	131	Malaysia
mdv	132	Maldives
mli	133	Mali
mlt	134	Malta
mrt	136	Mauritania
mus	137	Mauritius
mex	138	Mexico
mng	141	Mongolia
mar	143	Morocco
moz	144	Mozambique
mda	146	Republic of Moldova
nam	147	Namibia
nru	148	Nauru
npl	149	Nepal
nld	150	Netherlands (Kingdom of the)
ncl	153	New Caledonia
mkd	154	North Macedonia
vut	155	Vanuatu
nzl	156	New Zealand
nic	157	Nicaragua
ner	158	Niger
nga	159	Nigeria
niu	160	Niue
nor	162	Norway
pak	165	Pakistan
pan	166	Panama
cze	167	Czechia
png	168	Papua New Guinea
pry	169	Paraguay

per	170	Peru
phl	171	Philippines
pol	173	Poland
prt	174	Portugal
gnb	175	Guinea-Bissau
tls	176	Timor-Leste
pri	177	Puerto Rico
eri	178	Eritrea
qat	179	Qatar
zwe	181	Zimbabwe
rom	183	Romania
rwa	184	Rwanda
rus	185	Russian Federation
kna	188	Saint Kitts and Nevis
lca	189	Saint Lucia
vct	191	Saint Vincent and the Grenadines
stp	193	Sao Tome and Principe
sau	194	Saudi Arabia
sen	195	Senegal
syc	196	Seychelles
sle	197	Sierra Leone
svn	198	Slovenia
svk	199	Slovakia
sgp	200	Singapore
som	201	Somalia
zaf	202	South Africa
esp	203	Spain
sdn	206	Sudan (former)
sur	207	Suriname
tjk	208	Tajikistan
swz	209	Eswatini
swe	210	Sweden
che	211	Switzerland
syr	212	Syrian Arab Republic
tkm	213	Turkmenistan
twn	214	China, Taiwan Province of
tza	215	United Republic of Tanzania
tha	216	Thailand
tgo	217	Togo
tkl	218	Tokelau
ton	219	Tonga
tto	220	Trinidad and Tobago
omn	221	Oman
tun	222	Tunisia
tur	223	Turkiye
are	225	United Arab Emirates
uga	226	Uganda
tuv	227	Tuvalu
gbr	229	United Kingdom of Great Britain and Northern Ireland
ukr	230	Ukraine
usa	231	United States of America
bfa	233	Burkina Faso
ury	234	Uruguay
uzb	235	Uzbekistan
ven	236	Venezuela (Bolivarian Republic of)
vnm	237	Viet Nam
eth	238	Ethiopia
wsm	244	Samoa
yem	249	Yemen
cod	250	Democratic Republic of the Congo
zmb	251	Zambia
bel	255	Belgium
lux	256	Luxembourg
srb	272	Serbia
mne	273	Montenegro

Supplementary Table S3 | Comparison of T-FBS and FAO-FBS for dietary energy, fat, and protein supplies (2010–2022). The world total only includes countries available in the FAO-FBS dataset (see count). The mean, percentiles (5th, 50th/median, 95th), and standard deviation (SD) of the percentage differences between the T-FBS and FAO-FBS are summarized. This table provides supplementary information to Fig. 10 in the main text.

Year	Element	Count	FAO-FBS world (Ecal or Mt)	T-FBS world (Ecal or Mt)	Percentage diff w.r.t. FAO-FBS (%)				
					Mean (world)	5th	50th (median)	95th	SD
2010	Dietary energy	179	7154	7135	-0.3	-3	-0.10	7	11
2011	Dietary energy	179	7295	7278	-0.2	-2	-0.12	10	13
2012	Dietary energy	180	7441	7423	-0.2	-2	-0.12	12	12
2013	Dietary energy	180	7548	7529	-0.3	-3	-0.12	8	10
2014	Dietary energy	180	7723	7709	-0.2	-3	-0.09	6	8
2015	Dietary energy	180	7810	7796	-0.2	-2	-0.09	7	9
2016	Dietary energy	180	7922	7907	-0.2	-2	-0.07	6	8
2017	Dietary energy	180	8079	8063	-0.2	-3	-0.08	8	7
2018	Dietary energy	180	8193	8180	-0.2	-2	-0.11	5	7
2019	Dietary energy	185	8309	8299	-0.1	-3	-0.06	5	6
2020	Dietary energy	185	8412	8409	0.0	-4	-0.10	3	6
2021	Dietary energy	185	8533	8526	-0.1	-4	-0.15	1	6
2022	Dietary energy	185	8643	8639	0.0	-3	-0.10	3	7
2010	Fat	179	197	196	-0.4	-2	-0.13	12	24
2011	Fat	179	200	199	-0.4	-2	-0.14	13	20
2012	Fat	180	206	205	-0.4	-2	-0.15	10	20
2013	Fat	180	209	208	-0.4	-1	-0.14	11	23
2014	Fat	180	215	214	-0.3	-1	-0.10	12	18
2015	Fat	180	218	217	-0.2	-1	-0.14	13	21
2016	Fat	180	222	221	-0.3	-2	-0.12	11	21
2017	Fat	180	228	227	-0.3	-1	-0.10	14	19
2018	Fat	180	234	233	-0.3	-1	-0.10	8	17
2019	Fat	185	241	240	-0.1	-1	-0.07	11	13
2020	Fat	185	244	245	0.1	-2	-0.13	5	3
2021	Fat	185	251	251	-0.2	-1	-0.14	2	1
2022	Fat	185	253	253	-0.1	-1	-0.09	8	8
2010	Protein	179	214	214	-0.2	-1	0.25	5	4
2011	Protein	179	218	217	-0.2	-1	0.22	4	7
2012	Protein	180	222	221	-0.2	-1	0.24	4	6
2013	Protein	180	226	225	-0.2	-1	0.23	4	3
2014	Protein	180	231	230	-0.2	-1	0.28	4	2
2015	Protein	180	234	234	-0.2	-1	0.25	5	3
2016	Protein	180	239	239	-0.2	-1	0.25	3	2
2017	Protein	180	245	244	-0.2	-1	0.24	5	2
2018	Protein	180	248	248	-0.2	-1	0.21	4	2
2019	Protein	185	252	252	-0.1	-1	0.26	4	2
2020	Protein	185	257	257	0.0	-1	0.24	3	2
2021	Protein	185	261	261	-0.1	-1	0.25	2	3
2022	Protein	185	265	265	-0.1	-1	0.26	4	3

Supplementary Table S4 | Comparison between T-FBS and FAO-FBS for rice and products in 2022 in India. The table includes data for SUA commodities used for compiling the T-FBS. Since FAO-FBS does not provide opening and closing stocks, these elements are excluded from regional supply and regional demand calculations, and thus, the residuals calculation is adjusted to account for stock variation.

		**Rice and products (PCe)**			**SUA commodity**										
**Element**	**Unit**	**FAO-FBS**	**T-FBS**	**% diff.**	**Rice**	**Husked rice**	**Rice, milled**	**Rice, broken**	**Bran of rice**	**Rice, milled (husked)**	**Flour of rice**	**Starch of rice**	**Rice, gluten**	**Oil of rice bran**	**Cake of rice bran**
Production	1000 t	196246	196246	0%	196246	718	116141	5830	13930	0	120	228	29	918	5244
Import	1000 t	18	23	27%	1	1	15	0	0	0	0	0	0	0	0
Opening stocks	1000 t		46494		0	27	34000	0	0	0	0	0	0	677	0
**Regional supply**	1000 t	196264	242763		196247	745	150157	5830	13930	0	120	228	29	1595	5244
Food	1000 t	140276	125706	-10%	0	702	91457	1365	0	0	120	0	0	229	0
Feed	1000 t	12314	29647	141%	12313	0	0	0	7374	0	0	0	0	0	5244
Seed	1000 t	3275	3275	0%	3275	0	0	0	0	0	0	0	0	0	0
Loss	1000 t	5196	5196	0%	5196	0	0	0	0	0	0	0	0	0	0
Processed	1000 t	596	675	13%	175000	0	5830	571	6556	0	0	227	0	152	0
Other uses	1000 t	0	555		0	0	0	0	0	0	0	0	28	350	0
Export	1000 t	33107	30083	-9%	464	300	17866	3934	0	26	3	3	0	0	1
Closing stocks	1000 t		48099		0	27	35000	0	0	0	0	0	0	865	0
**Regional demand**	1000 t	194764	243237		196249	1029	150152	5870	13930	26	123	229	29	1595	5245
Residuals	1000 t	1	-475		-2	-283	4	-40	0	-26	-3	-1	0	0	-1
Stock variation	1000 t	1499	1605	7%	0	0	1000	0	0	0	0	0	0	187	0
Net export	1000 t	33089	30060	-9%	463	299	17850	3934	0	26	3	2	0	0	1
Dietary energy	Billion kcal	326819	328880	1%	0	2463	319184	4750	0	0	422	0	0	2061	0
Fat	1000 t	854	1083	27%	0	18	823	12	0	0	2	0	0	229	0
Protein	1000 t	6652	6652	0%	0	55	6493	96	0	0	7	0	0	0	0

Supplementary Table S5 | Comparison between T-FBS and FAO-FBS for maize and products in 2022 at the world level. The table includes data for SUA commodities used for compiling T-FBS. Since FAO-FBS does not provide opening and closing stocks, these elements are excluded from regional supply and regional demand calculations, and thus, the residuals calculation is adjusted to account for stock variation.

Element	Unit	Maize and products (PCe)			SUA commodity								
		FAO-FBS	T-FBS	% diff.	Maize (corn)	Flour of maize	Bran of maize	Maize gluten	Germ of maize	Starch of maize	Gluten feed and meal	Oil of maize	Cake of maize
Production	Mt	1162	1164	0.1%	1164	161	20	6	11	39	10	3	3
Import	Mt	229	225	-2%	210	3	2	0	0	2	6	1	0
Opening stocks	Mt		422		411	1	0	0	0	0	5	3	0
**Regional supply**	Mt	**1391**	**1811**		**1785**	**165**	**22**	**6**	**12**	**41**	**21**	**6**	**3**
Food	Mt	134	131	-2%	6	107	0	0	1	2	0	3	0
Feed	Mt	717	743	4%	703	0	14	2	4	0	10	0	3
Seed	Mt	8	8	0%	8	0	0	0	0	0	0	0	0
Loss	Mt	62	62	0%	62	0	0	0	0	0	0	0	0
Processed	Mt	68	32	-52%	224	49	5	4	6	22	0	0	0
Other uses	Mt	196	193	-2%	166	4	0	0	0	15	0	0	0
Export	Mt	219	225	2%	210	3	2	0	0	2	6	1	0
Closing stocks	Mt		409		398	1	0	0	0	0	5	3	0
**Regional demand**	Mt	**1404**	**1803**		**1777**	**165**	**22**	**6**	**12**	**41**	**21**	**6**	**3**
Residuals	Mt	0	7		8	0	0.1	0	-0.1	-0.1	-0.3	-0.1	0
Stock Variation	Mt	-13.3	-12.9	-3%	-13.3	0	0	0	0	0	0.1	0.1	0
Net export	Mt	-10	0		0	0	0	0	0	0	0	0	0
Dietary energy	Billion kcal	401838	433653	8%	19776	379073	0	85	3473	7597	0	23648	0
Fat	1000 t	3465	6187	79%	239	3222	0	0	98	4	0	2625	0
Protein	1000 t	8398	8624	3%	500	7839	0	21	253	11	0	0	0

Supplementary Table S6 | Comparison between T-FBS and FAO-FBS for wheat and products in 2022 at the world level. The table includes data for SUA commodities used for compiling T-FBS. Since FAO-FBS does not provide opening and closing stocks, these elements are excluded from regional supply and regional demand calculations, and thus, the residuals calculation is adjusted to account for stock variation.

Element	Unit	Wheat and products (PCe)			SUA commodity													
		**FAO-FBS**	**T-FBS**	**% diff.**	**Wheat**	**Wheat and meslin flour**	**Food preparations**	**Bulgur**	**Breakfast cereals**	**Bran of wheat**	**Germ of wheat**	**Wheat gluten**	**Starch of wheat**	**Bread**	**Pastry**	**Uncooked pasta**	**Wafers**	**Mixes and doughs**
Production	Mt	810	808	-0.2%	808	437	0	0	4	92	9	1	7	5	7	7	1	0
Import	Mt	320	231	-28%	188	14	4	0	3	8	0	1	1	1	13	5	2	2
Opening stocks	Mt	0	401		386	4	1	0	2	0	0	0	0	0	0	4	1	1
**Regional supply**	**Mt**	**1130**	**1441**		**1383**	**455**	**5**	**0**	**9**	**100**	**9**	**2**	**8**	**6**	**20**	**17**	**5**	**3**
Food	Mt	546	460	-16%	0.5	406	1	0	3	20	2	0	0	5	7	7	1	1
Feed	Mt	200	232	16%	150	0	0	0	0	72	7	0	1	0	0	0	0	0
Seed	Mt	34	34	0.1%	34	0	0	0	0	0	0	0	0	0	0	0	0	0
Loss	Mt	30	30	0%	30	0	0	0	0	0	0	0	0	0	0	0	0	0
Processed	Mt	13	3	-76%	549	29	0	0	0	0	0	0	2	0	0	0	0	0
Other uses	Mt	30	25	-19%	19	0	0	0	0	0	0	1	4	0	0	0	0	0
Export	Mt	252	231	-8%	188	14	4	0	3	8	0	1	1	1	13	5	2	2
Closing stocks	Mt		425		410	4	1	0	2	0	0	0	0	0	0	4	1	1
**Regional demand**	**Mt**	**1105**	**1440**		**1380**	**454**	**7**	**0**	**9**	**100**	**9**	**2**	**8**	**6**	**20**	**16**	**5**	**3**
Residuals	Mt	2	1		3	1	-2	0	0	0	0	0	0	0	0	0	0	-1
Stock variation	Mt	24	23	-1.5%	23	0	0	0	0	0	0	0	0	0	0	0	0	0
Net export	Mt	-68	0		0	0	0	0	0	0	0	0	0	0	0	0	0	0
Dietary energy	Billion kcal	1552711	1553028	0%	1552	1402066	1505	139	12786	54978	6048	204	725	14137	27503	23973	4525	2888
Fat	1000 t	9143	9148	0.1%	10	6498	11	1	87	920	171	3	1	176	1038	123	33	77
Protein	1000 t	50105	50116	0%	55	44270	47	5	337	3052	440	39	0	476	456	813	83	42
